# Endothelial extracellular vesicles promote tumour growth by tumour‐associated macrophage reprogramming

**DOI:** 10.1002/jev2.12228

**Published:** 2022-06-03

**Authors:** Makon‐Sébastien Njock, Tina O'Grady, Olivier Nivelles, Michelle Lion, Sophie Jacques, Maureen Cambier, Stephanie Herkenne, Florian Muller, Aurélie Christian, Claire Remacle, Julien Guiot, Souad Rahmouni, Franck Dequiedt, Ingrid Struman

**Affiliations:** ^1^ Laboratory of Molecular Angiogenesis GIGA Research Centre University of Liège Liège Belgium; ^2^ Laboratory of Gene Expression and Cancer GIGA‐MBD University of Liège Liège Belgium; ^3^ Laboratory of Animal Genomics GIGA‐Medical Genomics GIGA Research Centre University of Liège Liège Belgium; ^4^ Department of Pneumology GIGA Research Centre University and CHU of Liège Liège Belgium

**Keywords:** breast cancer, extracellular vesicles, macrophages, microRNA, TAM

## Abstract

Tumour‐derived extracellular vesicles (EVs) participate in tumour progression by deregulating various physiological processes including angiogenesis and inflammation. Here we report that EVs released by endothelial cells in a mammary tumour environment participate in the recruitment of macrophages within the tumour, leading to an immunomodulatory phenotype permissive for tumour growth. Using RNA‐Seq approaches, we identified several microRNAs (miRNAs) found in endothelial EVs sharing common targets involved in the regulation of the immune system. To further study the impact of these miRNAs in a mouse tumour model, we focused on three miRNAs that are conserved between humans and mouse, that is, miR‐142‐5p, miR‐183‐5p and miR‐222‐3p. These miRNAs are released from endothelial cells in a tumour microenvironment and are transferred via EVs to macrophages. In mouse mammary tumour models, treatment with EVs enriched in these miRNAs leads to a polarization of macrophages toward an M2‐like phenotype, which in turn promotes tumour growth.

## 1 INTRODUCTION

Cancer is a dynamic disease. From the initial conversion of a nonmalignant cell to a malignant cell, the developing tumour constantly evolves and generally becomes more heterogeneous during the course of the disease. Tumour heterogeneity includes not only genomic heterogeneity among neoplastic cells, but also heterogeneity of the noncancerous compartment of the tumour microenvironment (TME), including immune, mesenchymal and endothelial cells (ECs) as well as noncellular components. Communication within this complex environment is achieved through direct cell‐cell contact and also via secreted factors. Of these, extracellular vesicles (EVs), such as exosomes, are emerging as important TME intercellular communication mediators.

Extracellular vesicles are secreted by most, if not all, cell types present in the TME. The content of EVs is dependent on the type and physiological state of the producing cell and includes noncoding small RNAs, mRNAs, proteins and lipids. EVs have been classified according to their subcellular origin (Colombo et al., 2014). EVs directly formed and released from the cellular plasma membrane are called microparticles, microvesicles or ectosomes, and display a diverse range of sizes (100–1000 nm in diameter). Internal vesicles generated within multivesicular endosomal compartments are secreted into the extracellular environment when these compartments fuse with the plasma membrane: these EVs are termed exosomes. Exosomes are also defined by their size (50–150 nm) and their endosome‐associated protein content. In this work, we will use the general term “EV” to refer to small EVs that express specific exosomal markers.

Extracellular vesicles secreted by ECs are important players in endothelial function and contribute to angiogenesis‐related diseases such as cardiovascular disease and cancer. Numerous cancer studies have highlighted the proangiogenic properties of EVs secreted by tumour cells in various cancer types (for review, see (Théry et al., 2019)). Within the tumoural environment, the exchange of materials via EVs has been observed between tumour cells and ECs (Ludwig & Whiteside, 2018). For example, we previously showed that ECs shed miRNAs via EVs that can prevent tumour growth (Bovy et al., 2015). Conversely, ECs can also receive EVs that modulate their phenotype from other cell types present in their environment. For instance, mesenchymal stem cells (MSCs) have been found to secrete EVs that can promote angiogenesis *in vitro* and *in vivo* (Liang et al., 2016).

Until now, most studies have related EV communication involving only two cell types. In this work we report that EVs released by tumour‐exposed ECs participate in the recruitment of macrophages into the tumour, leading to an immunomodulatory phenotype permissive for tumour growth.

## 2 RESULTS

### 2.1 TME modulates endothelial phenotype

**FIGURE 1:**
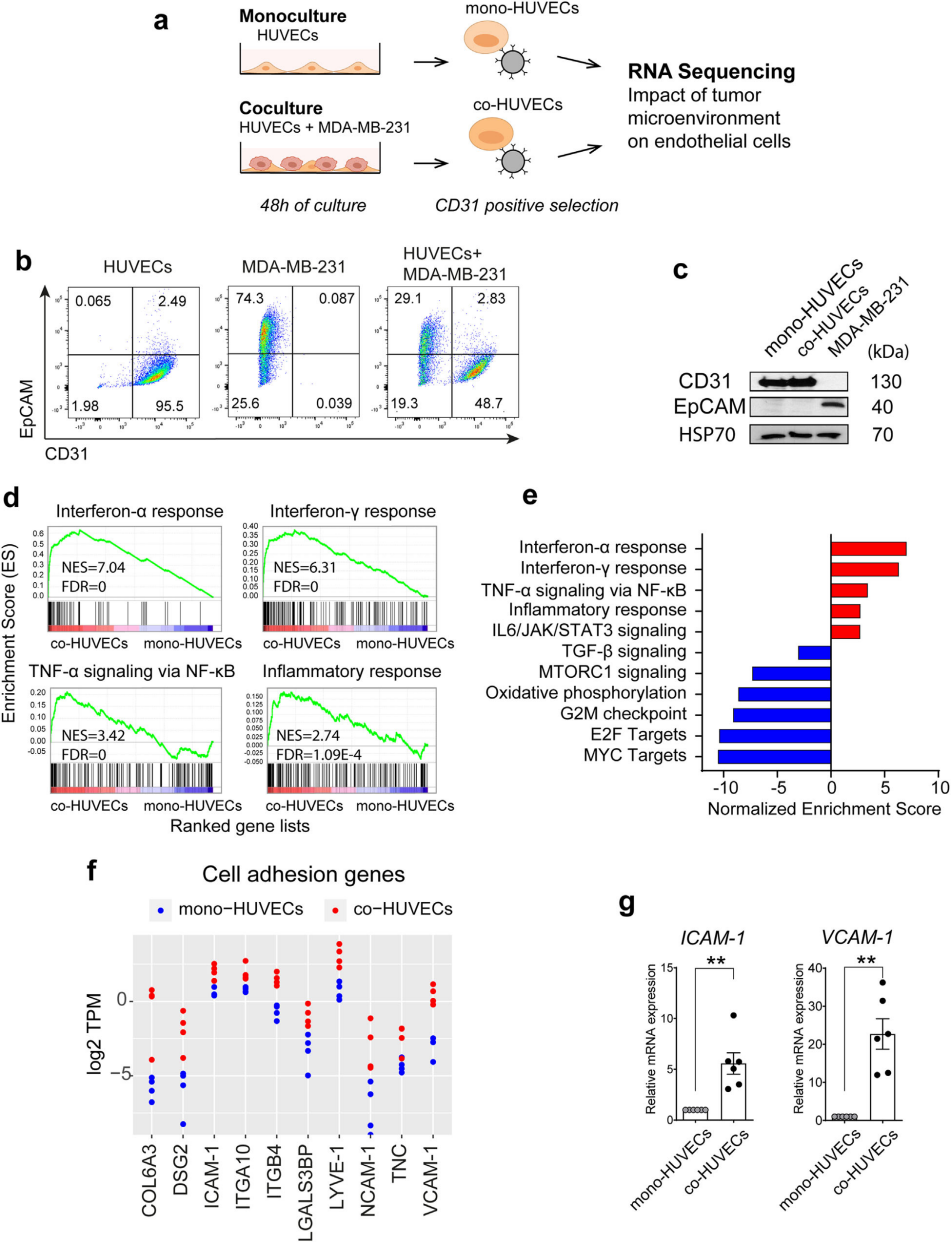
Co‐culture with cancer cells induces an activation of ECs via inflammatory signalling pathways. (a), Schematic of the *in vitro* coculture system used to study the effect of TME generated by human breast cancer cells (MDA‐MB‐231) on human endothelial cells (HUVECs). HUVECs were cultured with/without MDA‐MB‐231 for 48 h (i.e. coculture vs. monoculture), followed by endothelial cell isolation using magnetic beads coated with anti‐CD31 antibody. Then, RNA‐Seq was performed to assess transcriptomic changes induced by MDA‐MD‐231. (b), Flow cytometry analysis of HUVEC, MDA‐MB‐231 and cocultured HUVEC/MDA‐MB‐231 cells stained for the CD31 endothelial marker and EpCAM. (c), Western blotting of endothelial marker CD31 and breast cancer marker EpCAM in lysates from HUVECs purified from mono or cancer cell coculture and from MDA‐MB‐231 cells. HSP70 is a loading control. (d), Selected gene sets significantly enriched in co‐ versus mono‐HUVECs from GSEA of RNA‐Seq data obtained as in (a). (e), GSEA enrichment of inflammation‐associated pathways in co‐HUVECs. (f), Expression levels (log2TPM) of several adhesion molecules in HUVECs monocultures or in HUVECs cocultured with MDA‐MB‐231 as shown by RNA‐Seq data. TPM = Transcripts per Million. (g), RT‐qPCR analysis of expression of the adhesion molecules ICAM‐1, VCAM‐1 in HUVECs cocultured with MDA‐MB‐231 relative to HUVECs alone. Data are mean ± SEM. **P < 0.01 calculated by One‐Sample T‐test (*n* = 6)

To determine the impact of the TME on EC behaviour we sought to identify key pathways altered in ECs when exposed to tumour cells. To this aim, we used a coculture system in which human umbilical vein endothelial cells (HUVECs) were cultured together with human triple‐negative breast cancer cells (MDA‐MB‐231). After 48 h, mono‐ or cocultured HUVECs were purified using magnetic beads coated with antibodies to CD31 (Figure 1a), an endothelial marker that is universally expressed by HUVECs (98% by flow cytometry) but is near undetectable in MDA‐MB‐231 (Figure 1b). As expected, isolated cells showed enrichment of CD31 and absence of the breast cancer marker EpCAM by western blot (Figure 1c) and RT‐qPCR analysis (Figure S1a), confirming the purity of HUVEC preparations. Next, we isolated RNA from four independent EC preparations and performed long RNA sequencing (long RNA‐Seq)) (Figure S1b). Principal component analysis (PCA) of the RNA‐Seq data revealed that the repertoire of long RNAs in HUVECs from the coculture condition (co‐HUVECs) clustered together, but apart from that of HUVECs from the monoculture condition (mono‐HUVECs) (Figure S1c)).

GSEA revealed that several tumour‐associated pathways were affected in co‐HUVECs (Figure 1d). In particular, several inflammatory‐related gene sets were enriched, including interferon‐α and ‐γ responses (NES = 7.04, FDR = 0 and NES = 6.31, FDR = 0, respectively), TNF‐α signalling (NES = 3.42, FDR = 0), and Inflammatory response (NES = 2.74, FDR = 1.09E‐4) (Figure 1e). Up‐regulation of these pathways suggests that ECs have changed their inflammation status and indeed, several cell adhesion genes were up‐regulated in co‐HUVECs (Figure 1f). RT‐qPCR analysis confirmed induction of *Intercellular Adhesion Molecule 1* (*ICAM‐1*) and *Vascular Cell Adhesion Molecule‐1* (*VCAM‐1*) in the coculture condition (Figure 1g).

Overall these results indicate that coculture with tumour cells alters the EC transcriptome and induces activation via inflammatory signalling pathways.

### 2.2 TME modulates the content of endothelial EVs

**FIGURE 2:**
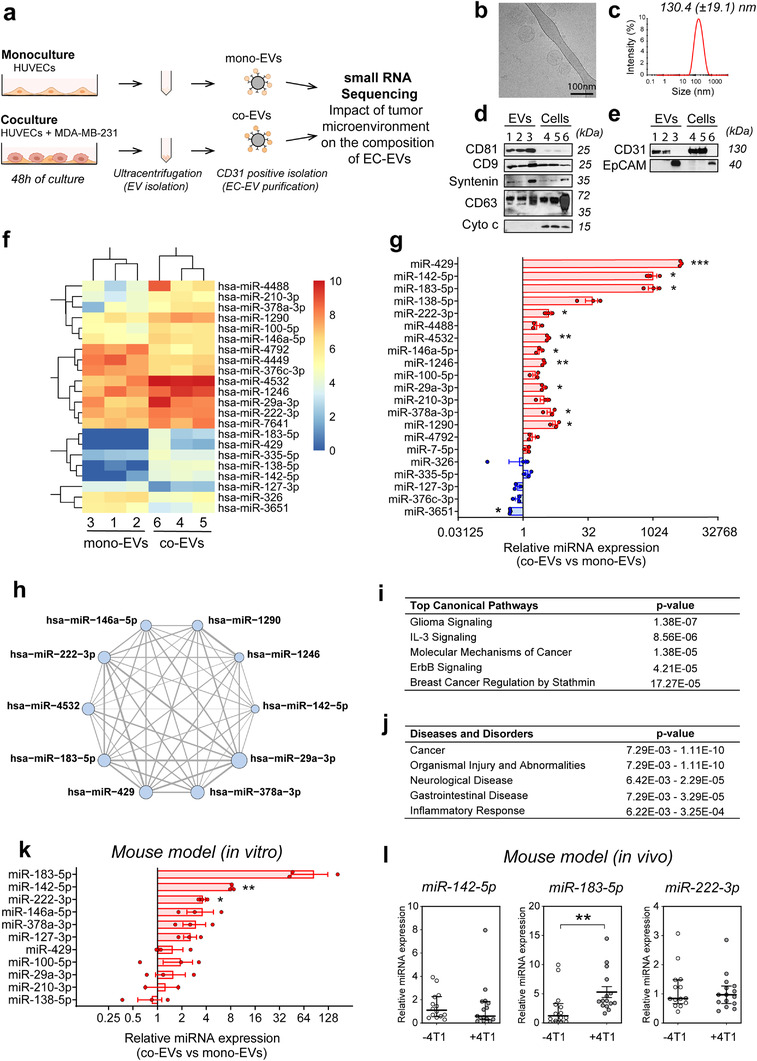
ECs release EVs with dysregulated miRNA levels when cultured in the presence of cancer cells, in particular miR‐142‐5p, miR‐183‐5p and miR‐222‐3p. (a), Overview of the procedure of endothelial EV isolation and small RNA profile analysis. b‐e, Characterization of HUVEC EVs by cryo‐transmission electron microscopy analysis bar represents 100 nm (b), dynamic light scattering analysis (c), and western blotting of the exosomal markers CD9, CD81, CD63 and syntenin in lysates from purified HUVEC EVs and cells from mono‐ and coculture conditions (d). EV lysates: 1: mono‐HUVEC EVs, 2: co‐HUVEC EVs, 3: MDA‐MB‐231‐EVs; cell lysates: 4: mono‐HUVECs, 5: co‐HUVECs, 6: MDA‐MB‐231. (e), Western blotting of endothelial marker CD31 and tumour marker EpCAM in lysates from purified HUVEC EVs and cells from mono‐ and coculture conditions. EV lysates: 1: mono‐HUVEC EVs, 2: co‐HUVEC EVs, 3: MDA‐MB‐231‐EVs; cell lysates: 4: mono‐HUVECs, 5: co‐HUVECs, 6: MDA‐MB‐231. (f), Heatmap of miRNAs with differential abundance between mono‐HUVEC EVs and co‐HUVEC EVs. (g), RT‐qPCR analysis of the miRNAs dysregulated in co‐HUVEC EVs. Data are mean ± SEM. *P < 0.05, **P < 0.01, ***P < 0.001 calculated by One‐sample t‐test (*n* = 3). (h), Gene targets shared by miRNAs with increased abundance in co‐EVs (nodes). Edge thickness is scaled by the number of genes targeted in common by the connected nodes. Node size is scaled by number of predicted targets. i, j, Top canonical pathways (i) and top diseases and disorders (j) by IPA analysis of genes targeted by at least five miRNAs upregulated in co‐EVs. k, RT‐qPCR analysis of miRNAs dysregulated in EVs from MS1 cells in coculture (with 4T1 mouse breast cancer cell line) versus mono‐culture conditions. Data are presented as mean ± SEM. *P < 0.05 and **P < 0.01 calculated by One‐sample t‐test (*n* = 3). l, Relative levels of miR‐142‐5p, miR‐183‐5p and miR‐222‐3p in endothelial EVs isolated from plasma of mouse model of 4T1 breast cancer compared to those from control, uninjected BALB/c mice. Data are presented as median ± IQR. **P < 0.01 calculated by unpaired two‐tailed Mann‐Whitney test. ‐4T1 (empty circle) *n* = 14; +4T1 (black circle) *n* = 15

After observing that tumour cells induced EC activation, we hypothesized that a tumoural context would also affect the cargo composition of EC‐derived EVs. Consequently, we sought to determine the repertoire of RNAs present in EVs released by ECs with or without exposure to cancer cells. To this aim, we collected culture supernatants from mono‐ and co‐HUVECs, isolated EVs via ultracentrifugation, and specifically retrieved endothelial EVs by affinity capture using anti‐CD31 magnetic beads (Figure 2a).

Analysis of the isolated vesicles by cryo‐transmission electron microscopy revealed a spherical morphology (Figure 2b and Figure S2a). Using dynamic light scattering, we found their average size to be 130 nm (± 19.1 nm), a size range typically considered to be that of exosomes (Figure 2c). These vesicles were also enriched for four classical exosomal markers, CD81, CD9, CD63 and syntenin. As expected, we did not detect mitochondrial Cytochrome C (Figure 2d), indicating that these vesicle preparations were enriched in exosomes. As we did for cells, we checked the purity of our endothelial EV preparations by assessing the level of the endothelial marker CD31 and the breast cancer marker EpCAM. Importantly, the enrichment of CD31 and the absence of EpCAM indicate that our EV preparations contain endothelial EVs that are not contaminated by EVs derived from tumour cells (Figure 2e). We then isolated total RNA from the monoculture‐derived HUVEC EVs (mono‐EVs) and co‐culture‐derived HUVEC EVs (co‐EVs). Because EVs are known to be enriched for small RNA fragments and a Bioanalyser analysis confirmed this to be true for our EV preparations (Figure S2b), we opted to profile the small RNA content of mono‐EVs and co‐EVs using RNA‐Seq. As expected, RNA‐Seq analysis revealed a wide variety of miRs in EVs. We detected 392 miRs with average normalized read counts of at least 1 in either mono‐EVs or co‐EVs (Table S1). The most abundant miRNA in both mono‐EVs and co‐EVs was miR‐126, an endothelial‐specific miRNA (Van Solingen et al., 2009), further establishing the effectiveness of our bead‐based endothelial purification method.

Most recent works have highlighted the role of miRNAs in the RNA function of EVs. To what extent other RNA species play a role in EV biology is still unclear. We thus decided to focus on the impact of TME on miRNA content of endothelial EVs. After mapping RNA‐Seq reads to miRNAs described in miRBase (http://www.mirbase.org)(Kozomara et al., 2019) and performing differential expression analysis, we identified 22 miRNAs that were significantly altered in co‐ versus mono‐EVs, with 16 and six being up‐ or down‐regulated, respectively, in EVs isolated from cocultured ECs (Figure 2f). RT‐qPCR largely confirmed these differences (Figure 2g). Using TargetScan (http://www.targetscan.org)(Agarwal et al., 2015), we found that many of the altered miRNAs shared common target genes (Figure 2h), suggesting that these miRNAs might be functionally linked. We created a list of genes targeted by at least five miRNAs increased in co‐EVs and used Ingenuity Pathways Analysis software to evaluate functional pathway enrichment. In these multitargeted genes, we observed significant enrichment in cancer‐associated pathways including Glioma Signalling (p = 1.38E‐07), IL‐3 Signalling (p = 8.56E‐06), Molecular Mechanisms of Cancer (p = 1.38E‐05), ErbB Signalling (p = 4.21E‐05), and Breast Cancer Regulation by Stathmin1 (p = 17.27E‐05) (Figure 2i), as well as enrichment in gene sets associated with Cancer and Inflammatory Response (Figure 2j).

To investigate whether these observations could be reproduced in another species, we repeated the coculture experiments using mouse ECs (MS‐1 cell line) and triple‐negative breast cancer cells (4T1 cell line). EVs purified from MS‐1 cells by ultracentrifugation showed enrichment of the exosome makers CD9, CD63, CD81 and syntenin (Figure S2c). As CD31 is also expressed in the mouse EC cell line (Figure S2d) we again used CD31‐based purification to isolate endothelial cells and EVs from mouse cocultures. We selected 12 miRNAs that were highly altered in human co‐EVs and have strong sequence conservation between human and mouse to assess by RT‐qPCR. The majority of these showed consistent regulation between humans and mouse (Figure 2k). On this basis, we tested our findings *in vivo*, in a mouse allograft model. We implanted 4T1 cells into the flanks of BALB/c mice and after 21 days isolated EVs specifically released by ECs into the bloodstream using an anti‐CD31 capture approach. Exosomal markers were readily detected in these EV preparations (Figure S2e). We chose to focus on the three miRNAs that were the most up‐regulated in our mouse *in vitro* assay, that is, miR‐142‐5p, miR‐183‐5p and miR‐222‐3p. While all three miRNAs were detected in CD31+ circulating EVs, only miR‐183‐5p appeared to be significantly up‐regulated in the circulation of 4T1‐injected mice compared to phosphate‐buffered saline‐injected control mice (Figure 2l).

Altogether, these results suggest that cancer cells modulate the release of specific miRNAs into endothelial EVs, and that many of these altered miRNAs are conserved between mouse and human.

### 2.3 Endothelial EVs generated in tumoural context are able to deliver miR‐142‐5p, miR‐183‐5p and miR‐222‐3p in macrophages

**FIGURE 3:**
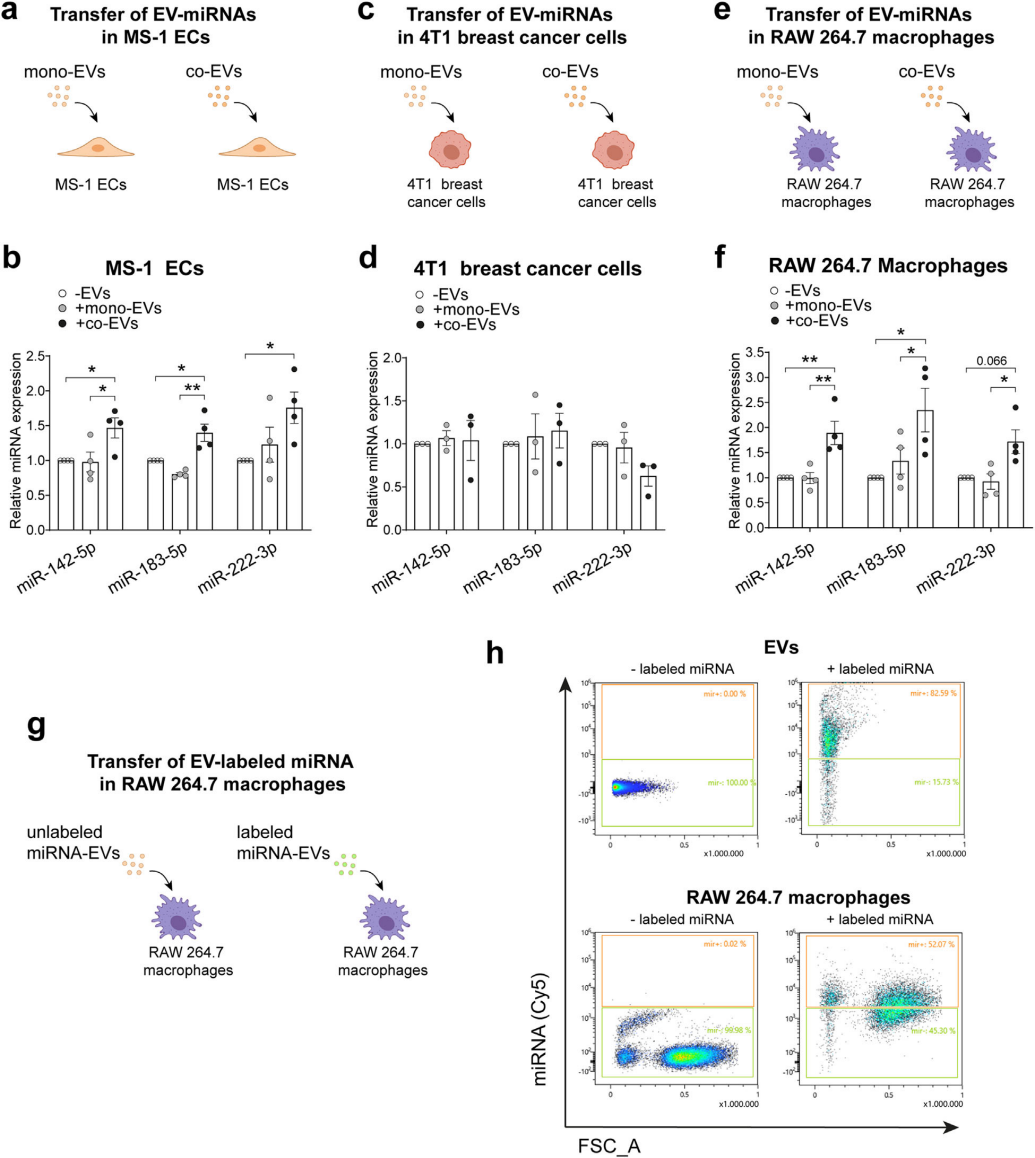
Endothelial EVs generated in the presence of cancer cells deliver miR‐142‐5p, miR‐183‐5p and miR‐222‐3p to RAW 264.7 macrophages. (a, c, e), Overview of the protocol to assess the transfer of miRNAs from co‐EVs to target cells. MS‐1 ECs (a), 4T1 breast cancer cells (c) or RAW 264.7 macrophages (e) were incubated for 24 h with endothelial MS‐1 EVs (3 μg/ml) purified from supernatants of MS‐1 alone (mono‐EVs) or MS‐1 cocultured with 4T1 mouse tumour cells (co‐EVs). (b, d, f), Relative levels of miR‐142‐5p, miR‐183‐5p and miR‐222‐3p in MS‐1 (b), 4T1 (d) or RAW 264.7 (f) cells treated with mono‐EVs and co‐EVs compared to those without EV treatment, assessed by RT‐qPCR. All data are mean ± SEM. RNA levels are expressed relative to the respective controls (‐EVs condition). *P < 0.05, **P < 0.01 and ***P < 0.001 calculated by one‐way ANOVA with Tukey's test (*n* = 3‐4). (g), Overview of the protocol to assess the transfer of Cy5‐miRNAs from EVs to macrophages. h, Flow cytometry analysis of Cy5‐miR‐183‐5p electroporated in MS‐1 EVs and incubated with RAW macrophages for 24h

Based on our observations so far, we postulated that increased packaging of specific miRNAs in endothelial EVs in response to tumour cells might have biological consequences in recipient cells. To test this model, we first sought to identify the cells in which these miRNAs are delivered. We decided to focus on three cell types: ECs, macrophages and cancer cells. We first isolated endothelial EVs from mouse MS‐1 ECs either cultured alone (mono‐EVs) or in the presence of 4T1 breast cancer cells (co‐EVs). Then, purified mono‐ or co‐EVs were incubated with mouse endothelial, cancer or macrophage cell lines (MS‐1, 4T1 or RAW 264.7, respectively) for 24 h (Figure 3a,c,e) and levels of miR‐142‐5p, miR‐183‐5p and miR‐222‐3p were assessed in each of the recipient cell populations by qPCR. Incubation with co‐EVs significantly increased the levels of miR‐142‐5p and miR‐183‐5p in MS‐1 cells (*P* < 0.05 and *P* < 0.01 compared to mono‐EVs, respectively) and RAW 264.7 macrophages (*P* < 0.01 and *P* < 0.05 compared to mono‐EVs, respectively) (Figure 3b,f). The level of miR‐222‐3p tended also to increase in MS‐1 cells and in RAW 264.7 macrophages (*P* < 0.05 compared to mono‐EVs) (Figure 3b,f). Interestingly, there was no detectable increase in the levels of these miRNAs in cancer cells treated with co‐EVs (Figure 3d). This may indicate a specific tropism of co‐EVs for ECs and macrophages but not cancer cells, or may simply reflect higher basal levels of the three miRNAs in 4T1 cancer cells.

To determine whether elevated levels of miRNAs in the recipient macrophages resulted from a direct transfer of miRNAs from endothelial EVs, we measured the levels of the corresponding miRNA precursors before and after incubation with mono‐ or co‐EVs. We found no significant changes in the levels of any of the pri‐miRs, suggesting that miR‐142‐5p, miR‐183‐5p and miR‐222‐3p are directly transferred from endothelial EVs to macrophages (Figure S3).

Additionally, we treated macrophages with endothelial EVs loaded with fluorescent‐labelled miR‐183‐5p (Figure 3g). Analysis by flow cytometry showed that macrophages efficiently take up the labelled miRNA from EVs (Figure 3h).

### 2.4 Altered endothelial EV‐derived miRNAs promote tumour growth by inducing a M2‐like polarization of tumour‐associated macrophages

**FIGURE 4:**
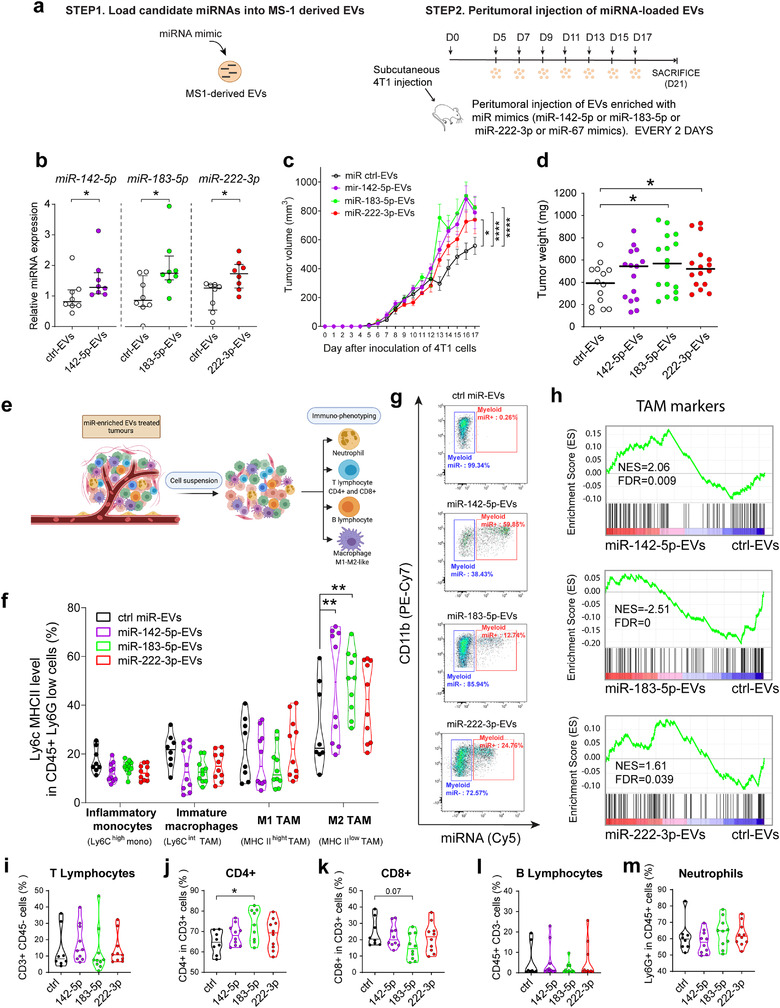
miR‐142‐5p, miR‐183‐5p and miR‐222‐3p increase tumour growth in a murine model of breast cancer by promoting M2‐like macrophage polarization. (a), Overview of the 4T1‐tumour bearing mouse model treated with EV‐enriched with miRNAs: endothelial EVs were loaded with miRNA mimics and injected every 2 days in mice harbouring 4T1 tumours. (b), Relative levels of miR‐142‐5p, miR‐183‐5p and miR‐222‐3p assessed by RT‐qPCR in tumours from mice injected with EVs loaded with corresponding miRNA mimic compared to those injected with Evs loaded with cel‐miR‐67 (ctrl) mimic. Data is presented as median ± IQR. *P < 0.05 calculated by unpaired two‐tailed Mann‐Whitney test. (c), Kinetics of tumour growth in mice treated with miR‐142‐5p‐EVs, miR‐183‐5p‐EVs or miR‐222‐3p‐EVs compared with those treated with cel‐miR‐67(ctrl)‐EVs. *P < 0.05 and ****P < 0.0001 calculated by two‐way ANOVA with Dunnett's test. (d), Tumour weight at sacrifice of miRNA‐EV‐treated mice. Tumour weight at sacrifice (16 days after the first EV injection). Black bars indicate median values. *P < 0.05 calculated by unpaired two‐tailed Mann‐Whitney test. Data shown are from two independent experiments. (e), Schematic diagram of isolation and characterization of immune cells from 4T1‐tumour, (f), Violin plots comparing the percentages of the four TAM subpopulations in 4T1‐tumour bearing mice treated with cel‐miR‐67 (ctrl)‐EVs, miR‐142‐5p‐EVs, miR‐183‐5p‐EVs and miR‐222‐3p‐EVs: inflammatory monocytes (Ly6C^high^MHCII^low^), immature macrophages (Ly6C^high^MHCII^high^), classically activated macrophages (M1) (Ly6C^low^MHCII^high^) and alternatively activated macrophages (M2) (Ly6C^low^MHCII^low^). Bars indicate median values. *P < 0.05 calculated by two‐way ANOVA followed by Dunnett correction, *n* = 8–10 mice per group. (g), Flow cytometry analysis of 4T1‐tumour bearing mice treated every other day with cel‐miR‐67 (ctrl)‐EVs, miR‐142‐5p‐Evs, miR‐183‐5p‐Evs or miR‐222‐3p‐Evs and with the corresponding Cy5‐miR‐Evs the day before sacrifice (control was treated with unlabeled ctrl‐Evs). (h), GSEA showed enrichment of TAM markers in 4T1‐tumours treated with miR‐142‐5p‐EVs, miR‐183‐5p‐EVs or miR‐222‐3p‐EVs compared to those treated with cel‐miR‐67 (ctrl)‐EVs. Briefly, cells from mouse 4T1‐tumors (treated with the different EV preparations) were collected, RNA‐Seq and GSEA analysis were performed (see Methods section for more details). *N* = 3 tumours per group. (i‐m), Quantification of flow cytometry analysis of T Lymphocytes (i), CD4+ (j) and CD8+ (k), B Lymphocytes (l) and neutrophils (m) in 4T1‐tumor bearing mice treated with EVs loaded with the indicated miRs. Bars indicate median values. *P < 0.05 calculated by one‐way ANOVA, *n* = = 8–10 mice per group. Gating strategy and flow cytometry analysis of corresponding graphs is shown in Figure S5 and S6

Having determined that the presence of cancer cells leads to enrichment of specific tumour‐associated miRNAs in EVs released by ECs, we next sought to investigate the impact of these miRNAs on tumour growth. To further examine their role in primary tumour growth, we injected tumour‐bearing mice peritumourally every 2 days with EC‐derived EVs electroporated with miR‐142‐5p, miR‐183‐5p, miR‐222‐3p or an exogenous miRNA not present in mice, cel‐miR‐67 (Figure 4a). An RNAse protection assay confirmed the efficiency of electroporation for miRNA loading into EVs (Figure S4a). As expected, cel‐miR‐67 was efficiently detected in the tumours after 19 days (Figure S4b). Treatment with EVs loaded with miR‐142‐5p, miR‐183‐5p or miR‐222‐3p also significantly increased the levels of the corresponding miRNAs in the tumours (*P* < 0.05) (Figure 4b). In contrast to the mice injected with cel‐miR‐67‐loaded ctrl‐EVs, mice treated with miR‐142‐5p‐EVs, miR‐183‐5p‐EVs or miR‐222‐3p‐EVs showed significant increases in tumour volume (*P* < 0.0001, *P* < 0.0001, *P* < 0.01, respectively) (Figure 4c). At sacrifice, the tumour weight was also significantly increased in mice treated with miRNA‐183‐5p‐ or miR‐222‐3p‐loaded EVs (*P* < 0.05) (Figure 4d). Importantly, treatment of tumour‐bearing mice by direct injection of miRNA mimics did not affect tumour growth, indicating that the miRNA needs to be encapsulated in EVs for its protumoural effect (Figure S5). Interestingly, combining miR‐142‐5p, miR‐183‐5p and miR‐222‐3p in EVs did not lead to any additive effects, suggesting that the miRNAs act via redundant pathways (Figure S6). Altogether, our data show that endothelial‐derived EVs loaded with mimics of miR‐142‐5p, miR‐183‐5p or miR‐222‐5p increase tumour growth in mice.

Because co‐EVs efficiently deliver miRNA to macrophages (Figure 3), we hypothesized that miRNAs present in EC‐derived EVs produced in the presence of cancer cells may promote tumour growth by modulating intratumoural infiltration or polarization of immune cells. Indeed, immunosuppressive immune cells such as tumour‐associated macrophages (TAMs), myeloid‐derived suppressor cells (MDSCs) or regulatory T (Treg) cells can promote tumour growth and metastasis by attenuating the immune response. To test this model, we evaluated the immune profile of 4T1 tumours following treatment with miR‐loaded EVs (Figure 4e; gating strategy is shown in Figure S7).

We first analysed myeloid cells, a major component of the immune cell repertoire present in the tumour. Treatment with miR‐142‐5p‐EVs, miR‐183‐5p‐EVs or miR‐222‐3p‐EVs did not affect the infiltration of neutrophils into 4T1 tumours (Figure 4m and Figure S8). To assess the impact of miRNA‐enriched EVs on TAMs, we classified them into five subpopulations based on the expression of Ly6C and MHCII(Movahedi et al., 2010) (Figure 4f and Figure S6a‐S8d): inflammatory monocytes (Ly6C^high^MHCII^low^), immature macrophages (Ly6C^high^MHCII^high^), classically activated (M1) macrophages (Ly6C^low^MHCII^high^) and alternatively activated (M2) macrophages (Ly6C^low^MHCII^low^) and other myeloid cells. Using this classification, M2 macrophages have been reported to be immunosuppressive and tumour‐promoting *in vivo*, whereas M1 macrophages are inflammatory and can eradicate tumours (see discussion). Interestingly, treatment with miR‐142‐5p‐EVs, miR‐183‐5p‐EVs, or miR‐222‐3p‐EVs increased the percentage of Ly6C^low^MHCII^low^ TAMs, indicating an M2‐like immunosuppressive phenotype (*P* < 0.05) (Figure 4f and Figure S8). Consistent with the functional impact of miR‐EVs on macrophage polarization, we observed that macrophages consistently took up EVs with labelled miRNA *in vivo* (Figure 4g), while few endothelial and tumour cells incorporated the EVs and no signal was observed in B or T cells (Figure S9).

To further analyse the impact of miRNA‐enriched EVs at the molecular level we performed RNA‐Seq transcriptomic profiling of 4T1 tumours treated with corresponding EVs. Although efforts have previously been made to characterize TAMs at the molecular level, a general signature is lacking (see discussion and (Cassetta et al., 2019)). GSEA analysis of the RNA‐Seq data with a TAM signature gene list (Tuit et al., 2019) revealed significant enrichment of TAM markers in 4T1 tumours treated with miR‐142‐5p‐EVs and miR‐222‐3p‐EVs compared to those treated with cel‐miR‐67 (ctrl)‐EVs (NES = 2.06, FDR q = 0.009; and NES = 1.61, FDR q = 0.039; respectively) and a depletion of TAM markers in 4T1 tumours treated with miR‐183‐5p‐EVs (NES = ‐2.51, FDR q = 0) (Figure 4h and marker from Table S2). In addition, GSEA analysis with M1 and M2 signature gene lists(Martinez et al., 2006) revealed that M1‐like macrophage gene markers were significantly depleted in 4T1 tumours treated with miR‐142‐5p‐EVs, miR‐183‐5p‐EVs and miR‐222‐3p‐EVs compared with those treated with cel‐miR‐67 (ctrl)‐EVs (NES = ‐2.35, FDR q = 6.54E‐4; NES = ‐1.92, FDR q = 6.6E‐3; NES = ‐1.87, FDR q = 0.011; respectively) (Figure S10a and table S2). In parallel, M2‐like macrophage gene markers were significantly enriched in 4T1 tumours treated with miR‐142‐5p‐EVs (NES = 1.44, FDR q = 0.07) (Figure S10b). Furthermore, treatment with miR‐142‐5p‐EVs and miR‐183‐5p‐EVs increased the expression of *arginase 1* (*ARG‐1*), a well‐known M2 marker, in the 4T1 tumours (*P* < 0.05, *P* < 0.01, respectively) (Figure S10c). In contrast, expression of *inducible nitric oxide synthase* (*iNOS*) (an M1 marker) was not modified by miRNA‐loaded EV treatment.

Next we further analysed the proportion of other immune cells present in the tumours and we found that treatment with miR‐142‐5p‐EVs, miR‐183‐5p‐EVs or miR‐222‐3p‐EVs did not modify the proportion of intratumoural T‐ or B‐lymphocytes (Figure 4i‐l and Figure S8a). Interestingly, treatment with miR‐183‐5p‐EVs led to a significant increase in the proportion of CD4+ and decrease in the proportion of CD8+ tumour‐infiltrating T lymphocytes (Figure 4j,k, Figure S8e) even though these cells did not incorporate the labeled‐miRNA EVs (Figure S9)

Altogether, our data show that endothelial‐derived EVs containing miR‐142‐5p, miR‐183‐5p, or miR‐222‐3p lead to an increase in TAM in tumours.

### 2.5 miR‐142‐3p, miR‐183‐5p and miR‐222‐3p induce M2 polarization by targeting PTEN

**FIGURE 5:**
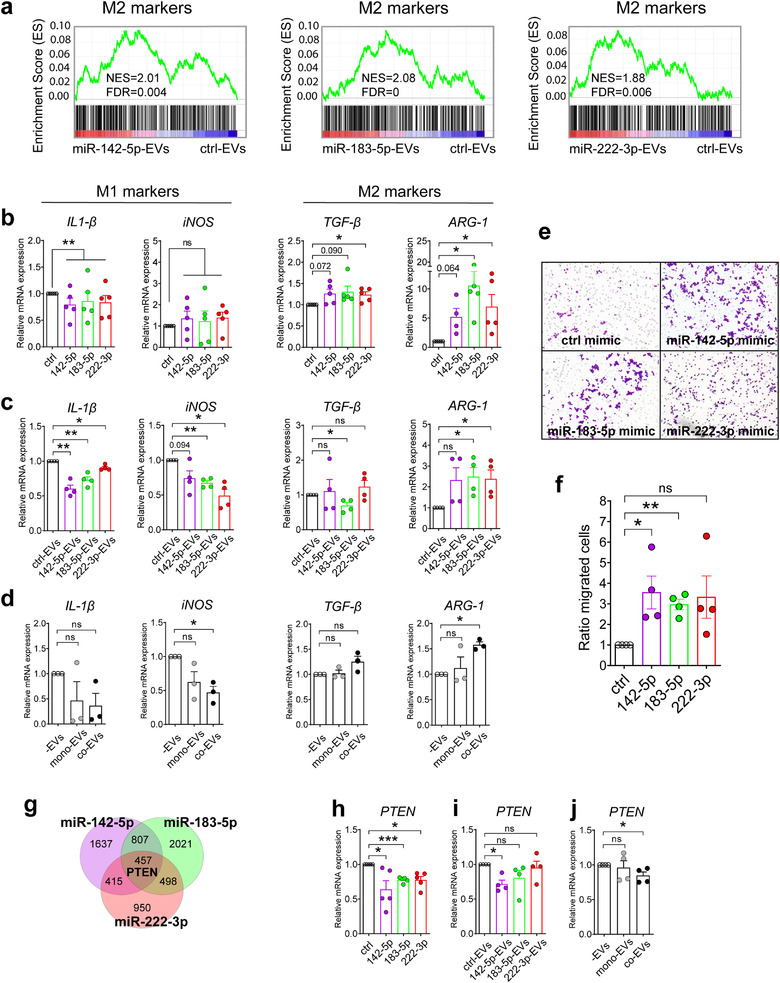
miR‐142‐5p, miR‐183‐5p and miR‐222‐3p alter the expression of M1/M2 markers of RAW 264.7 macrophages and increase their migration capacity. (a), Depleted/enriched gene sets associated with M2 markers in RAW 264.7 macrophages treated with miR‐142‐5p‐EVs, miR‐183‐5p‐Evs or miR‐222‐3p‐Evs compared to cel‐miR‐67(ctrl)‐Evs from ranked GSEA analysis. (b, c), Impact of miR‐142‐5p, miR‐183‐5p and miR‐222‐3p on the expression of M1 (*IL‐1β, iNOS*) and M2 markers (*TGF‐β, ARG‐1*) in RAW 264.7 macrophages (b) transfected with corresponding mimics (*n* = 5) or (c) treated with miRNA‐enriched EVs (*n* = 4), assessed by RT‐qPCR. (d), Relative expression of M1/M2 markers in RAW 264.7 macrophages treated with co‐ or mono‐EVs compared to those without EV treatment, assessed by RT‐qPCR (*n* = 3). (e, f), Impact of miR‐142‐5p, miR‐183‐5p and miR‐222‐3p on the migration of RAW 264.7 macrophages. (e), Representative images of transwell migration assay of RAW 264.7 macrophages transfected with cel‐miR‐67 (ctrl), miR‐142‐5p, miR‐183‐5p and miR‐222‐3p mimics. (f), Quantification of the migration of RAW 264.7 macrophages shown in e. Data are presented as mean ± SEM. *P < 0.05, **P < 0.01 calculated by One‐sample t‐test. (g), Venn diagram of mRNA targets of miR‐142‐5p (purple circle), miR‐183‐5p (green circle) and miR‐222‐3p (red circle). (h, i), Impact of miR‐142‐5p, miR‐183‐5p and miR‐222‐3p on the expression of their common target *PTEN* in RAW 264.7 macrophages (h) transfected with corresponding mimics (*n* = 5) or (i) treated with miRNA‐enriched EVs (*n* = 4), assessed by RT‐qPCR. (j), Impact of co‐ or mono‐EVs on the expression of *PTEN* in RAW 264.7 macrophages (*n* = 4). Data are presented as mean ± SEM. (b, c, f, h, i,j) relative to the respective controls (black bars).*P < 0.05, **P < 0.01, ***P < 0.001 calculated by One‐sample t‐test

In order to evaluate the impact of miR‐142‐5p, miR‐183‐5p and miR‐222‐3p on macrophage polarization, RAW 264.7 cells were treated with endothelial EVs transfected with miRNA mimics and the expression of M1 and M2 markers was assessed by RNA‐Seq. Because the *in vitro* M1/M2 gene response has been shown to differ from the *in vivo* gene response(Orecchioni et al., 2019), we compared gene expression levels in our dataset with gene response signatures established *in vitro* in peripheral blood mononuclear cells (PBMCs) activated classically by LPS+IFN‐γ or alternatively by IL‐4 (Table S2). We found an enrichment of M2‐like markers for cells treated with miR‐142‐5p‐, miR‐183‐5p‐ and miR‐222‐3p‐transfected EVs compared to those treated with EVs transfected with control miR mimic (NES = 2.01, FDR q = 4.0E‐3; NES = 2.08, FDR q = 0; NES = 1.88, FDR q = 6.0E‐3; respectively) (Figure 5a), while M1‐like markers were not affected (not shown). Interestingly, M2 markers have been shown to correlate with poor survival in cancer patients(Orecchioni et al., 2019). This signature was confirmed by orthogonally testing several canonical M1 and M2 markers by RT‐qPCR. Indeed, the levels of M2 markers *ARG‐1* and *TGF‐β* (*Transforming Growth Factor‐β*) increased in cells treated with miR‐142‐5p‐, miR‐183‐5p‐ and miR‐222‐3p‐transfected EVs, while the levels of M1 markers *iNOS* and *IL‐1β* (*Interleukin‐1β*) were not significantly affected (Figure 5b).

Next, we investigated the effect of miRNA‐enriched EVs on macrophage polarization. MiR‐142‐5p, miR‐183‐5p and miR‐222‐3p were efficiently delivered by EVs in RAW 264.7 cells (Figure S11a‐c) and led to an increase in the expression of *ARG‐1* (Figure 5c). Furthermore, treatment with miRNA‐enriched EVs also decreased the M1 markers *iNOS* and *IL‐1β*. To confirm the effects of miR‐142‐5p, miR‐183‐5p and miR‐222‐3p, we induced M2 polarization by treating RAW 264.7 macrophages with interleukin 4 and transfected the cells with antimiRNAs. In line with the results described above, knockdown of miR142‐5p, miR‐183‐5p or miR222‐3p increased the abundance of M1 markers *iNOS* and *IL‐1β*, while the M2 markers were not changed (Figure S12).

Based on the above observations, we anticipated that co‐EVs, which are enriched in miR‐142‐5p, miR183‐5p and miR‐222‐3p would also promote M2 polarization of recipient macrophages. To test this, we incubated RAW 264.7 cells with mono‐ and co‐EVs (3 μg/ml) for 24 h. While mono‐EVs had some effect on macrophage differentiation as previously described(Njock et al., 2015), the effect of co‐EVs was consistently more robust, inducing dysregulation of the two canonical markers: up‐regulation of *ARG‐1* mRNA (*P* < 0.05) and down‐regulation of *iNOS* mRNA (*P* < 0.05) (Figure 5d).

Many studies have reported that TAMs are either recruited from circulating macrophages or arise from differentiation of tissue‐resident macrophages (Cassetta et al., 2019; Arwert et al., 2018). Since we observed an increased number of M2‐like TAMs without a corresponding reduced level of other macrophage populations in the tumours (Figure 4f), we examined whether miRNAs enriched in co‐EVs could affect the migration of macrophages in a Boyden chamber assay. Strikingly, we observed a 3‐fold increase in the migration rate of macrophages transfected with miRNAs enriched in co‐EVs (Figure 5e,f).

To more deeply explore the mechanism by which co‐EV miRs increase the level of M2‐like macrophages in tumours, we searched for miRNA targets that may contribute to macrophage polarization. Using TargetScan we identified 457 common targets of the three miRNAs. Among them, we chose to validate PTEN, which is a known regulator of macrophage polarization (Wang et al., 2018) (Figure 5g). In addition, *PTEN* has been previously reported to be a target of miR‐142‐5p (Xu & Wang, 2018), miR‐183‐5p (Wang et al., 2019), and miR‐222‐3p (Li et al., 2016), which we confirmed by transfecting miRNA mimics into macrophages (Figure 5h) and with incubation of macrophages with transfected endothelial EVs (Figure 5i) followed by assessment of PTEN levels in treated cells with RT‐qPCR. More importantly, treatment with co‐EVs also decreased PTEN mRNA expression in macrophages (Figure 5j).

Collectively, our data show that miR‐142‐5p, miR‐183‐5p or miR‐222‐5p mimics lead to a shift toward M2 polarization of macrophages *in vitro*, potentially via the regulation of their common target, *PTEN*.

### 2.6 miR‐142‐3p, miR‐183‐5p and miR‐222‐3p are elevated in breast cancer patients while PTEN is decreased

**FIGURE 6:**
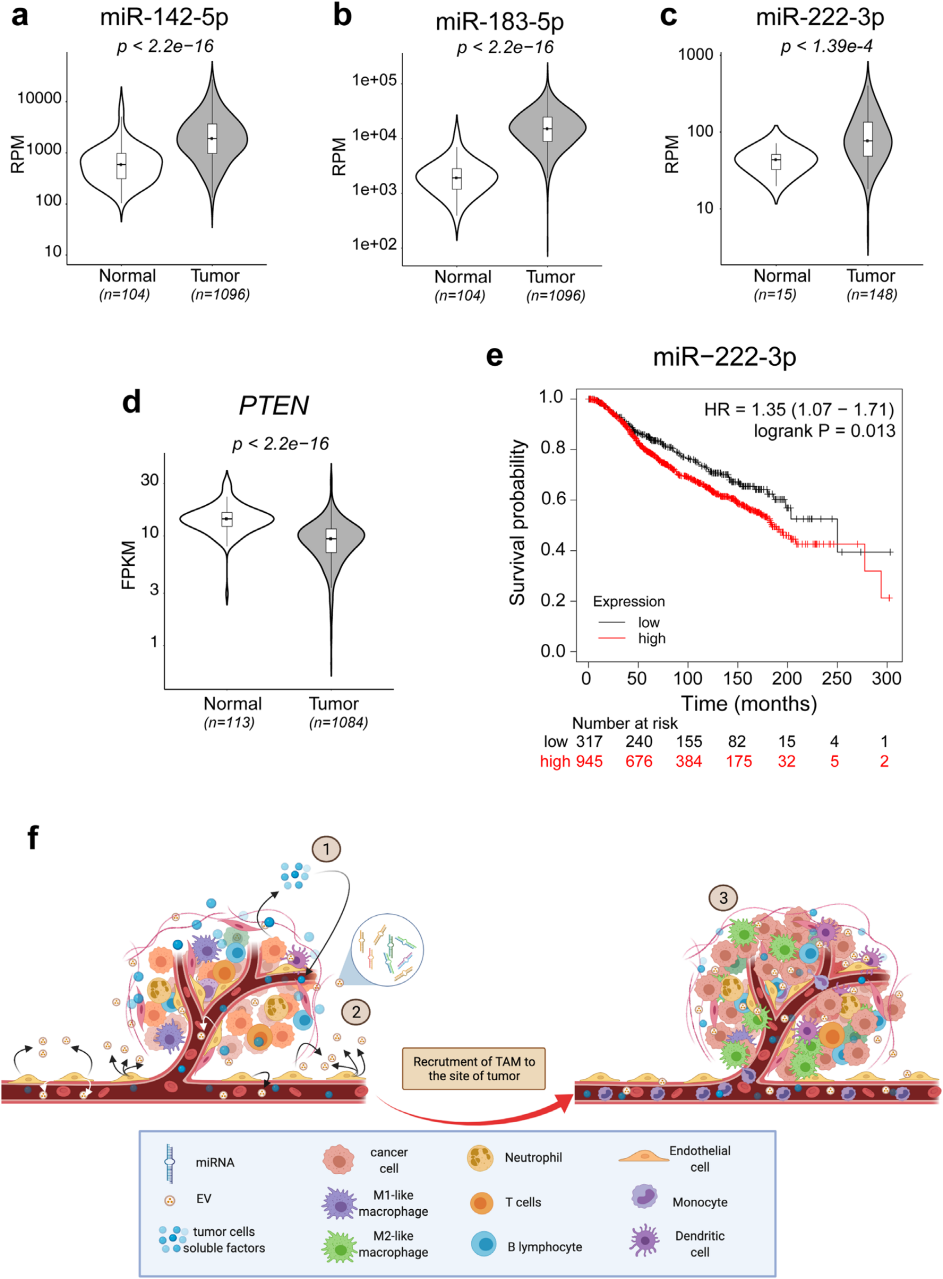
miR‐142‐3p, miR‐183‐5p and miR‐222‐3p are elevated in breast cancer patients while PTEN is decreased. (a,b), Levels of miR‐142‐5p (a) and miR‐183‐5p (b) in normal tissue and tumours from breast cancer patients. RPM = RNA‐Seq reads per million mapped reads. (c), Levels of miR‐222‐3p in normal tissue and tumors from patients with basal‐like/ER(‐) breast cancer. RPM = RNA‐Seq reads per million mapped reads. (d), Levels of *PTEN* mRNA in normal tissue and tumours from breast cancer patients. FPKM = Fragments Per Kilobase of transcript per Million mapped RNA‐Seq reads. (e), Kaplan‐Meier survival plot of breast cancer patients with high vs. low levels of miR‐222. Expression range of the probe: 6–13, cutoff value: 7.52. (f), Model of how endothelial EVs promote tumor progression by inducing M2‐like polarization of TAMs. (1) In the presence of TME, ECs are activated and release EVs that contain several tumour‐associated miRNAs, including miR‐142‐5p, miR‐183‐5p and miR‐222‐3p. (2) These modified EVs deliver miR‐142‐5p, miR‐183‐5p and miR‐222‐3p into TAMs and induce M2‐like polarization. (3) An increased number of M2 TAMs promotes progression of the tumour

The above results suggest that in an experimental model of breast cancer, ECs are activated by tumour cells and increase secretion of several miRNAs, notably miR‐142‐5p, miR‐183‐5p and miR‐222‐3p, via packaging into EVs. These miR‐loaded EVs collectively reprogram the macrophages within the tumour to an M2‐like phenotype by targeting *PTEN* (Figure 6f).

Consistent with this model, the expression of miR‐142‐5p and miR‐183‐5p are significantly higher in the tumour tissue of patients with breast cancer than in healthy control tissue (Figure 6a,b). This regulation in not observed for miR‐222‐3p in a general cohort (Figure S13a). MiR‐222‐3p belongs to the miR‐221/222 miRNA cluster located on chromosome X, which is implicated in many cancers in women including ovarian, cervical, endometrial and breast cancers (Song, An et al., 2019). In the latter it has been shown to be correlated with aggressiveness (Stinson et al., 2011) and involved in chemoresistance (Li et al., 2020). Importantly, in ER+ breast cancer miR‐221/222 targets *ESR1*/ERα and can thereby confer resistance to estrogen/ERα‐targeted therapies. When we examined the basal‐like/ER(‐) breast cancer subtype, we found an elevated level of miR‐222‐3p in tumour tissue compared to healthy control tissue (Figure 6c). As expected, PTEN levels are lower in tumours from breast cancer patients (Figure 6d). In agreement with the above‐mentioned association of miR‐221/222 with aggressiveness, and our own findings of miR‐222‐3p‐EVs leading to increased tumour volume (Figure 4d), Kaplan–Meier survival analysis indicated a decreased survival rate for breast cancer ER+ patients with high levels of miR‐222 (Figure 6e). Increased levels of miR‐142‐5p and miR‐183‐5p were not found to be associated with decreased survival rate in breast cancer patients (Figure S13b,c).

## 3 DISCUSSION

Recently, a number of studies have shown that exosomes released by tumour cells can direct macrophage and neutrophil polarization to an M2 phenotype, thus promoting tumour progression (Wang et al., 2018; Park et al., 2019; Cooks et al., 2018). For instance, tumour cells in hypoxic environments release EVs that activate macrophages toward an M2 phenotype, thereby promoting the migration, invasion, and epithelial‐mesenchymal transition of pancreatic cancer cells (Wang et al., 2018). However, there has been virtually no evidence so far that exosomes of endothelial origin can also affect macrophage polarization and tumour progression. This is surprising since ECs are known to influence macrophage polarization in the context of vascular diseases such as arteriosclerosis (He et al., 2018). Here, we purified EVs from ECs cultured in the presence of breast cancer cells and explored the role and underlying mechanisms of endothelial EV RNA content in the progression of breast cancer. We unravelled an intricate mechanism by which cancer cells reprogram the RNA content of EVs released by intratumoural ECs, which drives polarization of macrophages toward an M2‐like phenotype, in turn promoting tumour growth (Figure 6f). Because exosomes can alter cellular functions via cargo miRNAs, we examined the levels of miRNAs in exosomes produced by ECs in a mammary tumour environment and identified several miRNAs sharing a series of common targets involved in the regulation of the immune system.

TAMs, a major component of TME, exhibit multiple protumoural activities, such as stimulation of tumour angiogenesis and tumour cell metastasis (Cassetta & Pollard, 2018). Previously, macrophages have been classified into two populations: fully polarized M1, or classically activated, macrophages arising from stimulation by microbial agents or proinflammatory factors such as lipopolysaccharides (LPS); and M2, or alternatively activated, macrophages arising in response to anti‐inflammatory molecules such as interleukin‐4 (IL‐4). In the tumour environment, M1‐ and M2‐like macrophages were originally described as having anti‐ and protumoural activities, respectively (Martinez et al., 2006). It is now known that TAMs display a pronounced heterogeneity and phenotypic plasticity, and tumours do not usually harbour macrophages with clearly defined sets of M1 or M2 markers. For instance, single‐cell analysis of myeloid cell infiltrate in breast cancer did not support this M1/M2 dichotomy in human cancers, but rather the presence of several clusters of TAMs expressing different levels of M2 markers in a continuous phenotypic spectrum (Azizi et al., 2018; Song, Hawkins, et al., 2019; Qian et al., 2020). Although some canonical markers such as iNOS and ARG‐1 may represent the two extremes of the M1 and M2 polarization states, macrophages with intermediate polarization and different activation markers often coexist in tumours. Furthermore, comparison of profiles between *in vitro*, mouse and human samples has failed to highlight a common M1/M2 signature (Martinez et al., 2006; Orecchioni et al., 2019). In our *in vitro* studies, we observed an enrichment of M2‐like signature genes, while we did not find enrichment of any M1 signature marker (Figure 5).

Our results indicate that EVs secreted by ECs in a tumour environment lead to an increase in the level of TAMs (Figure 4). This could result from increased local recruitment of M2‐like macrophages or imbalance in macrophage polarization at the tumour site. In addition, one could suggest a hybrid scenario, in which TAMs could be derived from circulating monocytes or from normal breast‐resident macrophages, which would then locally acquire specific transcriptomic profiles (Cassetta et al., 2019). Single‐cell RNA sequencing data lends evidence in favour of local recruitment (Qian et al., 2020; Song, Hawkins et al., 2019). Our data show no changes in the numbers of tumour‐associated monocytes, immature macrophages or M1‐like macrophages. Instead, we observed an increase in the M2‐like population, suggesting that TAMs are probably differentiated from circulating monocytes that are recruited into the tumour sites (Figure 4). The promigratory activity of the miRNAs enriched in co‐EVs that we observed *in vitro* is also in agreement with this hypothesis (Figure 5e,f). However, this issue deserves to be further investigated.

Although our study focused on EVs released by ECs in close proximity to tumour cells and acting at the tumour site, the observation that miR‐142‐5p (Ozawa et al., 2020) and miR‐222‐5p (Rodríguez‐Martínez et al., 2019) are elevated in circulating EVs in patients with breast cancer suggests that those encapsulated miRNAs could play additional roles at long distances. Several studies have shown that exosomes might be involved in metastasis (Wortzel et al., 2019). Consistent with these findings, recent publications have shown that EV‐derived miR‐142‐5p remodels lymphatic vessels to promote immune privilege in the TME (Zhou et al., 2021). Whether the elevated levels of exosomal miR‐142‐5p and miR‐222‐3p influence metastasis in breast cancer patients remains to be determined.

The exact contribution of EV miRNA in reprogramming recipient cells is still under debate. Because EVs contain a large variety of miRNAs and the number of molecules of any given miRNA is likely small, it is unlikely that a single EV‐derived miRNA could on its own regulate recipient cells (Chevillet et al., 2014). Here, we identified 22 miRNAs that show altered levels in CD31+ EVs when ECs are cocultured with cancer cells (Figure 2). One possible limitation of the study is that, although all EC express CD31, not all EVs release by endothelial cells might express this marker. Our findings are thus restricted to the CD31+ population of EVs produced by endothelial cells. A search for putative common targets of those miRNAs revealed many candidate genes, suggesting that miRNAs encapsulated in EVs might collectively regulate the same targets in recipient cells. Our observation that the combination of several miRNAs does not strengthen tumour‐promoting effects when compared to individual miRNAs is consistent with multiple miRNAs acting on the same targets. We also speculate that these miRNAs, when conserved between humans and mouse, participate in a similar system in a mouse cancer model. Although many studies have demonstrated that single miRNAs conserve their cancer‐regulatory functions between mice and humans, whether this is also the case for a pool of regulatory miRNAs has not been widely investigated. To our knowledge, our study is the first to highlight trans‐species conservation of the functions of a significant set of miRNAs in EVs in a disease model.

In order to exert their mRNA‐repressive function miRNAs must be delivered to recipient cells. We thus scrutinized the transfer of miRNAs in three potential recipient cell types in the TME. Importantly, myeloid cells captured many more fluorescent miRNAs *in vivo* than other cell types. *In vitro*, incubation of ECs or macrophages with EC‐derived EVs isolated from tumour‐associated coculture led to higher levels of miR‐222‐3p, miR‐142‐5p and miR‐183‐5p in both cell types (Figure 3). To rule out the possibility that EVs increased the intracellular levels of those miRNAs by stimulating their transcription, we measured the levels of the miRNAs precursors (pre‐ and pri‐miRNAs) in the recipient cells. We observed that miRNA precursors levels were not affected, supporting the idea that miR‐222‐3p, miR‐142‐5p and miR‐183‐5p are indeed directly transferred from EVs to ECs and macrophages. In addition, in a similar experimental set‐up we did not observe any increase of the miRNAs in tumour cells, indicating that EVs might target ECs and macrophages. Interestingly, PTEN, a common target for miR‐222‐3p, miR‐142‐5p and miR‐183‐5p, plays a role in protumoural M2 polarization, highlighting a functional response to macrophage uptake of tumour EC‐derived EVs (Li et al., 2015). The functional consequences of tumour EC‐EV uptake by ECs, on the other hand, remain to be elucidated.

In summary, our study has uncovered a sophisticated communication pathway within the TME: ECs, activated by cancer cells, release EVs that induce a local increase in TAMs and promote tumour growth (Figure 6f). Although EV transfer is not the only mechanism by which tumour cells modulate their microenvironment, our results uncover an important EV‐modulated communication network between the three main TME cell types. Endothelial EVs appear to represent important components of the TME and can act as significant messengers that mediate cross‐talk between the endothelium, TAMs and cancer cells. Targeting TAMs directly or manipulating their polarization is being evaluated as a new therapeutic strategy in cancer (Cassetta & Pollard, 2018). Our work provides evidence that modulating endothelial EVs to shape macrophage polarization represents a potential therapeutic approach.

## 4 METHODS

### 4.1 Design of the synthetic miRNA

The miRNA mimics miR‐142‐5p, miR‐183‐5p, miR‐222‐3p and cel‐miR‐67 (control) are double‐stranded RNAs designed using the method of Betancur et al. (2012). Briefly, the mature miRNA strand is modified by the addition of phosphorylation at the 5′ end and the carrier strand is the complementary RNA sequence, carrying a two base 3′ overhang with mutations near the 3′ end to thermodynamically destabilize the strand and induce faster degradation. AntimiRs have been designed to be fully complementary to the miR sequences (Lennox and Behlke, 2010). They are DNA/LNA mixmers with complete phosphorotioate backbones. LNAs were introduced to have a Tm between 70 and 75°C.

Tables S4 and S5 shows the sequence and modifications of the miRNA mimics and antimiRNAs. Oligonucleotides were purchased from Eurogentec.

### 4.2 Cell culture and co­culture experiments

Human Umbilical Vein Endothelial Cells (HUVECs) were cultured in endothelial growth media‐2 (EGM‐2, Lonza) with 5% FBS. Cells were used between passages 3–8. Human MDA‐MB‐231 breast cancer cells were cultured in Dulbecco's Modified Eagle Medium (DMEM) with 5% FBS. Mouse MS‐1 endothelial cell line, mouse 4T1 mammary carcinoma cell line and mouse RAW 264.7 macrophage cell line were cultured in DMEM with 10% FBS. All cells were cultured in EV‐depleted FBS (depleted by ultracentrifugation at 110,000×g for 16 h).

For coculture experiments, HUVECs were plated onto 10 T‐175 flasks (half confluence) and cultured in complete EGM‐2. In parallel, MDA‐MB‐231 cells were plated into 5 T‐175 flasks in DMEM with 10% FBS (EV‐depleted). After 48 h, HUVEC and MDA‐MD‐231 cells were rinsed with DPBS and placed in endothelial basal medium (EBM‐2) without standard growth factors but with 0.5% FBS (EV‐depleted) + Fibroblast Growth Factor (bFGF, 5 ng/ml) overnight. The next day, HUVECs were rinsed twice in DPBS and placed in EBM‐2 media with FBS (0.5%, EV‐depleted) + bFGF (5 ng/ml). MDA‐MB‐231 cells were trypsinized, rinsed with PBS and added to plates of adherent HUVECs in a 1:1 ratio. After 48 h, EVs were purified from the conditioned media of coculture (HUVEC+MDA‐MB‐231) or monoculture (HUVEC alone) flasks as described below. The protocol was the same for coculture of mouse MS1 endothelial cells with mouse 4T1 breast cancer cells with corresponding medium (DMEM with 10% FBS (EV‐depleted) on Day 1 and DMEM with 0.5% FBS (EV‐depleted) on Day 3.

### 4.3 Isolation of EVs from culture medium or plasma

First, conditioned medium was precleared by centrifugation at 400×g for 5 min, then 2,000×g for 20 min at 4°C, followed by centrifugation at 12,000×g for 45 min at 4°C. Then, the supernatants were passed through a 0.22‐μm filter (Millipore). To isolate EVs, culture medium was ultracentrifuged at 110,000×g for 120 min at 4°C, followed by washing of the pellet with PBS at 110,000×g for 120 min at 4°C (Optima XPN‐80 Ultracentrifuge, Beckman Coulter, SW32 rotor). The supernatant was discarded and the pellet was resuspended in PBS.

For coculture experiments, for human cells, endothelial EVs were purified and separated from cancer EVs with Dynabeads CD31 Endothelial Cell magnetic beads (#11155D, Invitrogen). CD31+ EVs were purified by resuspending the first ultracentrifuged EV pellet in Dynabeads Isolation buffer, adding 50 μl of washed beads per T‐175 of culture, incubating overnight at 4°C with rotation, washing twice with Isolation buffer, then resuspending beads with attached EVs in lysis buffer or PBS as required for downstream applications. Cells and EVs from monoculture endothelial cell samples were submitted to the same bead‐based CD31 purification. The protein levels of the EV preparations were measured using the BCA Protein Assay kit (Pierce) following the manufacturer's instructions. EVs were characterized by Dynamic light scattering, western blotting and electron microscopy.

Mouse MS1‐derived EVs and mouse EC‐derived EVs from plasma (500 μl) were first purified by ultracentrifugation (as described above in an Optima TL‐100 benchtop ultracentrifuge, TLA‐55 rotor) and CD31+ EVs were isolated using streptavidin mouse CD31 magnetic beads . To prepare these beads, 250 μl streptavidin magnetic beads (#S1420S, BioLabs) were washed 3 x 5 min with PBS with 0.1% BSA and incubated with 1 ml of antirat biotin‐conjugated capture antibody (#712‐065‐150, Jackson ImmunoResearch, 1/500) for 1 h. Beads were washed 3 x 1 min in PBS with 0.1%BSA. The beads were incubated for 2 h with 1 ml of rat antimouse CD31 antibody (#557355, BD Pharmingen, 1/200), followed by 3 x 1 min washes in PBS with 0.1 % BSA, then resuspended in PBS with 0.1% BSA. To purify mouse CD31+ EVs from supernatants (co‐ and mono‐conditions) or from mouse plasma (mouse with/without tumour), EV pellets from the first ultracentrifugation were incubated with 40 μl of streptavidin mouse CD31 magnetic beads overnight at 4°C with rotation, washing twice with PBS with 0.1 % BSA buffer, then resuspending beads with attached EVs in lysis buffer. For functional tests, EVs were eluted from magnetic beads with biotin elution buffer (2 mM) (#B4501, Sigma‐Aldrich).

### 4.4 Electroporation of EVs

Cel‐miR‐67 (ctrl)‐EVs, miR‐142‐5p‐EVs, miR‐183‐5p‐EVs and miR‐222‐3p‐EVs were prepared from MS‐1 EVs by eletroporating with the corresponding miRNA mimics. EVs were prepared from MS‐1 culture as described above from 20 subconfluent T‐175 flasks. The protein levels of the EV preparations were measured using the BCA Protein Assay kit (Pierce) following the manufacturer's instructions. 120 μg of EVs were electroporated with 20 μl of miR‐mimic (100 μM) in 500 μl of PBS using a Gene Pulser II at 400 V, 250 μF (Bio‐Rad). For the preparation of the miR‐mix, 120 μg of EVs were electroporated with 6.6 μl each of miR‐142‐5p‐mimic, miR‐183‐5p mimic and miR‐222‐3p mimic (all at 100 μM). The same procedure was used to prepare the Cy5‐labeled miR‐EVs.

### 4.5 RNAse treatment of EVs

Electroporated EVs were purified by ultracentrifugation as described above and purified EVs (10 μg) were subjected to 5 μg/ml RNAse A (#EN0531, Thermo Fisher Scientific), in a final volume of 50 μl, for 30 min at 37°C. As control, EV preparations were pre‐treated with 1% SDS 1%, 0.1% triton X‐100 for 10 min at RT. Preparations were then resuspended in Qiazol and purified as described below using the miReasy purification method (Qiagen).

### 4.6 Dynamic light scattering

EVs were suspended in PBS at a concentration of 50 μg protein/ml, and analyses were performed with a Zetasizer Nano ZS (Malvern Instruments, Ltd.). Intensity, volume and distribution data for each sample were collected on a continuous basis for 4 min in sets of three.

### 4.7 Western blotting

For EVs, sample lysis was performed using exosome lysis buffer (1% Triton, 0.1% SDS). Samples were denatured by incubation at 95°C for 7 min in 1x loading buffer. Equal amounts of protein lysate (5 μg) were electrophoresed on 12% SDS‐polyacrylamide gels and transferred to a polyvinylidene fluoride membrane using a wet transfer system. The blots were then blocked with either 5% BSA or commercial powdered milk at 8% for 1 h and then incubated overnight at 4°C with the primary antibody: CD9 (#sc20048, Santa Cruz), CD63 (#10628D, Invitrogen), CD81 (#10630D, Invitrogen), syntenin (#ab19903, Abcam), cytochrome c (#556433, BD pharmingen), CD31 (#M0823, DAKO), EpCAM (#93790S, Cell Signalling), or HSP70 (#SC33575, Santa Cruz). After three washes with TBS with 0.1% Tween‐20, membranes were incubated for 1 h at room temperature with an HRP‐conjugated secondary antibody (antirabbit (#7074S, Cell Signalling) or antimouse (#7076S, Cell Signalling)) before being revealed with enhanced chemiluminescence (ECL) substrate (Pierce Biotechnology).

### 4.8 Cryo‐transmission electron microscopy (Cryo‐TEM)

A 3 μl droplet of each EV suspension was applied to a glow discharged holey carbon grid (Lacey Carbon Grids). After the application of the suspension the grid was blotted against filter paper, leaving a thin sample film spanning the grid holes. These films were vitrified by plunging the grid into ethane, which was kept at its melting point by liquid nitrogen, using a Vitrobot (Thermo Fisher) to keep the sample at 95% humidity before blotting and freezing. The vitreous sample films were transferred to a Tecnai Arctica microscope (Thermo Fisher). Images were taken at 200 Kv with a field emission gun using a Falcon III (Thermo Fisher) direct electron detector.

### 4.9 Quantitative RT‐qPCR

For quantification of miRNA expression, 50 ng RNA was reverse transcribed into cDNA using qScript miRNA cDNA Synthesis kit (Quanta Biosciences), and qRT‐PCR was conducted in triplicate using Perfecta SYBR Green Super Mix (Quanta Biosciences). Thermal cycling was performed on an Applied Biosystems 7900 HT detection system (Applied Biosystems). In EVs, the relative miRNA levels were normalized to three internal controls selected from miRNA‐Seq analysis: miR‐191‐5p, miR‐26a‐5p and let‐7a‐5p, using the Delta‐Delta Ct method. For cells, fold changes were normalized to miR‐16‐5p and U6 small nuclear RNA.

For quantification of mRNA expression, 500 ng RNA was transcribed into cDNA using iScript cDNA Synthesis (Bio‐Rad), and qRT‐PCR was conducted in triplicate using Takyon MasterMix (Eurogentec). Fold changes were normalized to Glyceraldehyde 3‐phosphate dehydrogenase (*GAPDH*) using the Delta–Delta‐Delta Ct method. All qRT‐PCR primers are listed in Table S4.

### 4.10 Functional analysis of EVs 5000

RAW 264.7 macrophages, MS1 cells or cancer 4T1 cells were seeded in 12‐well plates. After 24 h, co‐EVs or mono‐EVs were added to cells at 3 μg protein/ml. 24 h later, cells were washed twice with PBS, then lysed to study miRNA or gene expression. For miRNA‐enriched EVs, RAW 264.7 macrophages were treated with miR‐67 (ctrl)‐EVs, miR‐142‐5p‐EVs, miR‐183‐5p‐EVs or miR‐222‐3p‐EVs (3 μg protein/ml) for 48 h.

### 4.11 Migration assay

Transfected RAW 264.7 cells were seeded in the upper wells of a Boyden chamber for assessment of chemotaxis toward medium containing 20% FCS in the lower chamber. The cells were allowed to migrate for 24 h at 37°C before staining with crystal violet and quantification.

### 4.12 Transfection of cells

Transfection of miRNA mimics: 200,000 cells were transfected in 6‐well plates with premiRs (12.5 nM), or antimiRs (10 nM) using DharmaFECT 4 transfection reagent (T‐2004‐03, Thermo Scientific) and analysed after 48 h. All miR mimics and antimiRs are listed in Table S3.

### 4.13 *In vivo* tumour models and treatments

Female BALB/c mice (6–8 weeks old) were purchased from Janvier Laboratories. In the mouse model of breast cancer, 1 × 10^5^ 4T1 cells were subcutaneously injected or not into the right flank of female BALB/c mice. Five days later, mice were randomly divided into four groups and had peritumoural injection of cel‐miR‐67 (ctrl)‐EVs, miR‐142‐5p‐EVs, miR‐183‐5p‐EVs or miR‐222‐3p‐EVs (3 μg/injection, every 2 days). On day 21, the mice were sacrificed to analyse the impact of miR‐142‐5p‐EVs, miR‐183‐5p‐EVs and miR‐222‐3p‐EVs on immune cell composition of the tumour (by flow cytometry) and molecular pathways (by RNA‐Seq). Subcutaneous tumour growth was recorded as the length (L) and width (W) of tumours by vernier calipers, and the tumour size (V) was calculated by the formula V = (L × W2)/2. The growth of subcutaneous tumours and survival of tumour‐bearing mice was monitored daily. The same procedures were performed using the same dose of miR‐mimics not encapsulated in EVs (0.5 μl of miR‐mimic (100 μM)/injection/ every 2 days).

To study the expression of candidate miRNAs in EC‐EVs from mouse plasma, BALB/c mice were subcutaneously injected or not with 4T1 cells (1 × 10^5^ 4T1 cells). After 21 days, CD31+ EVs were purified from plasma of mice with/without 4T1 cancer and miRNA levels were assessed by qRT‐PCR.

### 4.14 Flow cytometry

For flow cytometric analysis of tumour tissues, single cell suspensions were first prepared. The tumour masses were harvested from mice, cut into small pieces, and digested in DMEM medium containing collagenase A (1 mg/ml) (#10103586001, Roche) and collagenase D (1 mg/ml) (#11088866001, Roche) at 37°C for 30 min, and were then filtered with 70‐mm cell strainers (Becton and Dickinson).

The cells were stained with fluorescence‐labelled antibodies: PE‐anti‐CD3e (#553064), BV421‐anti‐CD45.2 (#560697), BUV395‐anti‐NK.1.1 (#564144), APC‐Cy7‐anti‐CD11c (#561241), AmCyan‐anti‐MHC‐II (#562366), BUV661‐anti‐CD4 (#612974), BV786‐anti‐CD8 (#563332). (all from BD Biosciences); FITC‐anti‐Ly 6C (#MCA2389A488) and APC‐anti‐CD206 (#MCA2235A647T) from (Bio‐Rad AbD Serotec); anti‐F4/80 (#123113), PE‐Texas Red‐anti‐Ly‐6G (#127647) were from Biolegend, PerCP‐Cy5.5‐anti‐CD11b (#45‐0112‐80) was from eBiosciences, , FVs510 Viability Staining Solution was used to exclude dead cells. Acquisition was performed on the BD FACSFortessa (BD Biosciences) and analysed using FlowJo (BD Biosciences) software.

### 4.15 Flow cytometric analysis of Cy5‐labeled miRs in tumour tissues

Tumour masses were harvested from mice, dissociated with a Gentlemacs instrument (Miltenyi), and digested in DMEM medium containing 1 mg/ml collagenase IA (C2674, Sigma) and 0.02 mg/ml DNAse (D4513, Sigma) at 37°C for 30 min (program 37C_m_LPDK), then filtered with 70‐mm cell strainers (Becton and Dickinson), centrifuged and resuspended in PBS. Resuspended cells were counted by cytometry. The cells were stained with fluorescence‐labelled antibodies in buffer (PBS with 1% BSA, 5 mM EDTA, 0.01% NaN3): FITC‐anti‐Ly6G (#127605, Biolegend), PE‐anti‐CD3 (#553064, BD), PE‐Cy7‐anti‐CD11b (#101215, Biolegend), APC‐Fire750‐anti‐Epcam (#118230, Biolegend), V450‐anti‐CD45.2 (#560697, BD), BV650‐anti‐CD45R‐B220 (#103241, Biolegend), PE‐Dazzle594‐anti‐CD31 (#102429, Biolegend) and Zombie Yellow (#423103, Biolegend), acquisition was performed on the SONY ID7000 (SONY) and analysed using Sony software.

### 4.16 Flow cytometric analysis of cell lines

Cells were trypsinized, washed and resuspended in PBS. 200,000 cells were stained with anti‐CD31‐FITC (Biolegend 303103), anti‐CD31‐PE‐Dazzle (#102429, Biolegend) or mEpCAM (#15237477, Thermo), hEpCAM (#324201, Biolegend) and PE‐antimouse (#P852, Thermo) in buffer (PBS with 1% BSA, 5 mM EDTA, 0,01% NaN3) and incubated 20 min at 4°C. Acquisition was performed on the BD FACSFortessa (BD Biosciences) and analysed using FlowJo (BD Biosciences) software

### 4.17 RNA library preparation and sequencing

RNA size profiles were determined with a Bioanalyser instrument. Long RNA libraries of mono‐HUVECs and co‐HUVECs were prepared for sequencing using the Ovation SoLo Human RNA‐Seq system (NuGEN) and sequenced as 75‐nt single end reads on an Illumina NextSeq500 instrument. Short RNA libraries (miRNA‐Seq) were prepared for sequencing using the SMARTer smRNA‐Seq Kit for Illumina (Clontech) and sequenced as 75‐nt single end reads on an Illumina NextSeq500 instrument. Long RNA libraries of RAW 264.7 cells and 4T1 tumours were prepared for sequencing using the Illumina TruSeq Stranded mRNA kit and sequenced as 151‐nt paired‐end reads on a NextSeq500 instrument.

### 4.18 Long RNA‐Seq analysis

For the long RNA‐Seq of mono‐HUVECs and co‐HUVECs (Ovation SoLo libraries), reads were filtered using Prinseq to trim the first 5 nt as per vendor protocol and to remove low‐complexity reads. Only reads of at least 10 nt after trimming were retained. Reads were then aligned to RefSeq human rRNA sequences with STAR v.2.5.2b and rRNA‐mapping reads were discarded. NonrRNA reads were then mapped with Salmon v.0.8.2 to Ensembl 90 cDNA and ncRNA. An in‐house script was used to remove PCR duplicates identified by mapping location and Ovation SoLo barcodes, and nonduplicated reads were mapped again with Salmon to the Ensembl 90 transcriptome. Finally, mRNA and lncRNA transcript abundance estimates were summed to the gene level using tximport. Differential expression was assessed and principal component analysis (PCA) was performed using DESeq2. For the mRNA‐Seq of RAW 264.7 cells and 4T1 tumours (Illumina TruSeq mRNA libraries), reads were mapped with Salmon v.0.8.2 to Ensembl 90 cDNA and ncRNA, then transcript abundance estimates were summed to the gene level using tximport and differential expression was assessed using DESeq2. Gene set enrichment analysis (GSEA) was performed on gene lists ranked by fold change (miR‐loaded EVs vs. control cel‐miR‐67‐loaded EVs) with GSEA software using the Hallmark gene sets or custom gene sets as indicated.

### 4.19 miRNA‐Seq analysis

RNA‐Seq reads from short RNA libraries were filtered and trimmed using Cutadapt according to Clontech recommendations (adapters and the first 3 nt were removed, and only reads 15 nt or longer were retained). Using mirDeep2 v.0.0.8 reads were mapped to the human genome (GRCh38) and reads mapping to miRNAs in miRBase were quantified. Differential abundance of miRNAs was assessed with DESeq2. MiRNAs with an adjusted *P*‐value of < 0.1 were considered to be differentially abundant between mono‐EVs and co‐EVs.

### 4.20 miRNA target analysis

Predicted human and mouse miRNA targets were downloaded from TargetScan 7.1. Only targets with cumulative weighted context++ score < 0 were considered. For IPA analysis, genes targeted by five or more of the 16 miRNAs increased in co‐EVs with TargetScan cumulative weighted context++ score < ‐0.1 were analysed with IPA canonical analysis (QIAGEN).

### 4.21 Analysis of miRs in breast cancer patients

TCGA‐BRCA miRNA and mRNA expression datasets were downloaded from the GDC Data Portal (https://portal.gdc.cancer.gov/). TCGA‐BRCA clinical information was downloaded from LinkedOmics (http://www.linkedomics.org/data_download/TCGA-BRCA/). RPM (for miRNA) or FKPM (for mRNA) values were plotted and compared using independent 2‐group Mann–Whitney U Tests in R.

Survival curves were plotted using METABRIC data with the Kaplan–Meier Plotter (kmplot.com), allowing cut‐off auto‐selection.

### 4.22 Statistical analysis

All experiments were performed at least three times unless otherwise stated. Data is shown as individual data points, mean ± standard error of the mean (SEM), or median ± interquartile range (IQR). Statistical analysis was performed using parametric two‐tailed Student's t‐test or nonparametric two‐tailed Mann–Whitney test for pairwise comparisons using GraphPad Prism software. For multiple comparisons, statistical analysis was performed using one (or two) ‐way ANOVA with Dunnett's test (or with Tukey's test). For all figures, *, **, *** and **** indicate *P* < 0.05, *P* < 0.01, *P* < 0.001 and *P* < 0.0001 comparisons between groups.

## CONFLICTS OF INTEREST

Nothing to declare.

## AUTHOR CONTRIBUTIONS

Makon‐Sébastien Njock designed, supervised and conducted *in vitro* and *in vivo* studies, statistical analyses, and interpreted the data. Tina O'Grady designed and performed bioinformatics analyses and interpreted the data. Makon‐Sébastien Njock and Tina O'Grady wrote the manuscript and designed the figures. Olivier Nivelles performed *in vivo* and *in vitro* studies. Michelle Lion and Maureen Cambier performed in vitro studies. Sophie Jacques and SR performed flow cytometry on tumour samples and analysed the corresponding data. Stephanie Herkenne performed *in vivo* studies. Florian Muller performed flow cytometry analysis. Aurélie Christian and Claire Remacle performed *in vitro* studies for the revision of the manuscript. Julien Guiot contributed to the experimental analysis and interpretation of the data. Ingrid Struman and Franck Dequiedt conceived and designed the study, coordinated the experiments, and wrote the manuscript. All authors read and approved the final manuscript.

## Supporting information

Supporting Information

Supporting Information

Supporting Information

Supporting Information

## Data Availability

RNA‐Seq data has been deposited in NCBI GEO under the accession numbers GSE167751, GSE167752 and GSE167753.
