# Supplementary material for: Endothelial extracellular vesicles promote tumour growth by tumour‐associated macrophage reprogramming
Source: J Extracell Vesicles. 2022 Jun 3;11(6):e12228. doi: 10.1002/jev2.12228 (PMC9164145; doi:10.1002/jev2.12228)
Supplement: Supplementary file 1 — Supporting Information [file JEV2-11-e12228-s004.pdf]

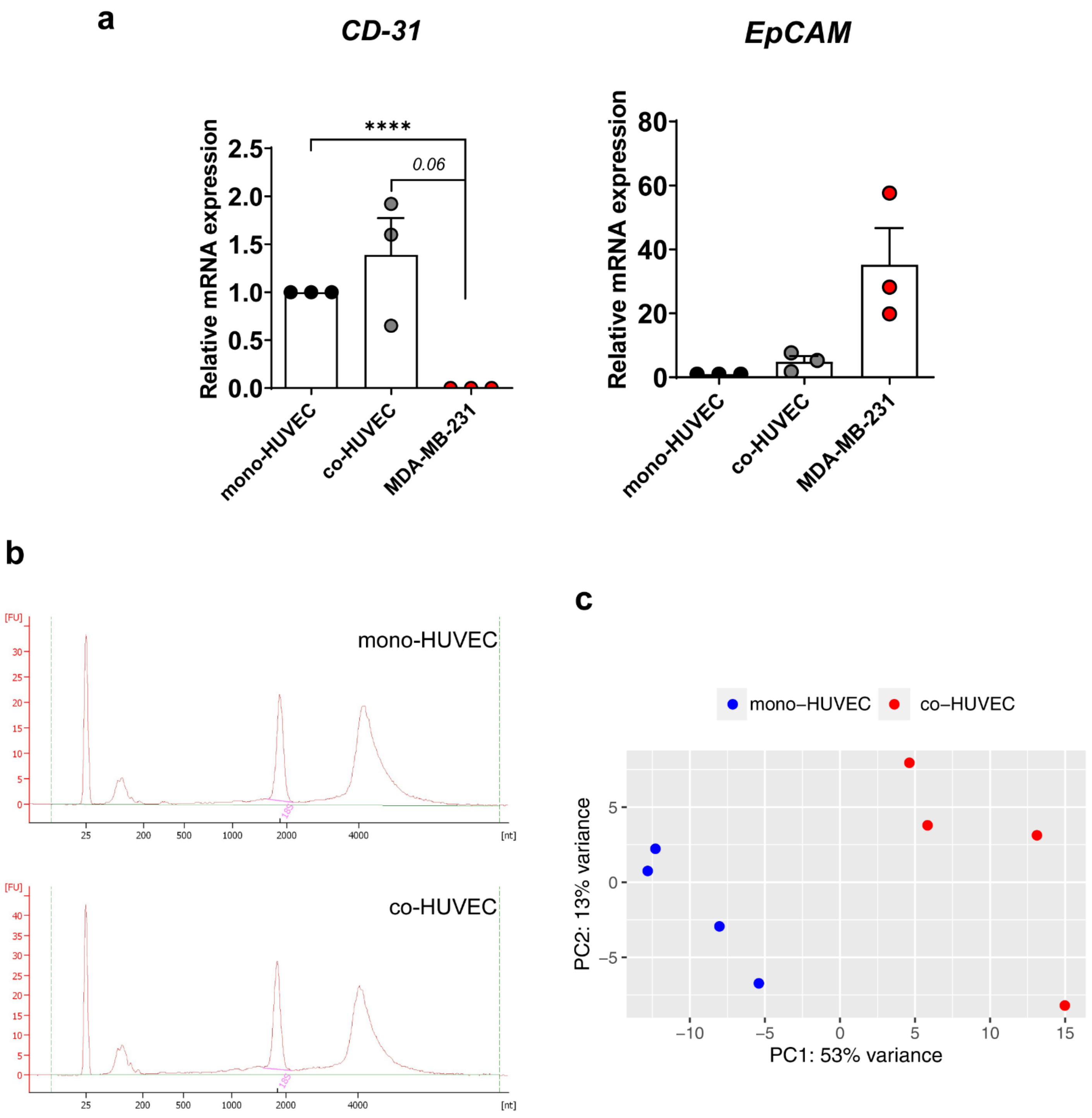

Supplementary Figure 1. Co-culture characterization at RNA level. a, HUVECs were cultured with/without MDA-MB-231 for 48 h (i.e. co-culture vs monoculture), followed by endothelial cell isolation using magnetic beads coated with anti-CD31 antibody. Bars shows RNA level of endothelial marker CD31 and breast cancer marker EpCAM in lysates from HUVECs purified from mono or cancer cell co-culture and from MDA-MB-231 cells, assessed by RT-qPCR. Data are presented as mean ± SEM. relative to the respective controls (black bars). **b,** Bioanalyzer analysis of total RNAs extracted from HUVECs cultured with/without MDA-MB-231 for 48 h (i.e. co-culture vs monoculture), followed by endothelial cell isolation using magnetic beads coated with anti-CD31 antibody. **c,** PCA plot of long RNA expression in HUVECs from coculture and monoculture conditions.

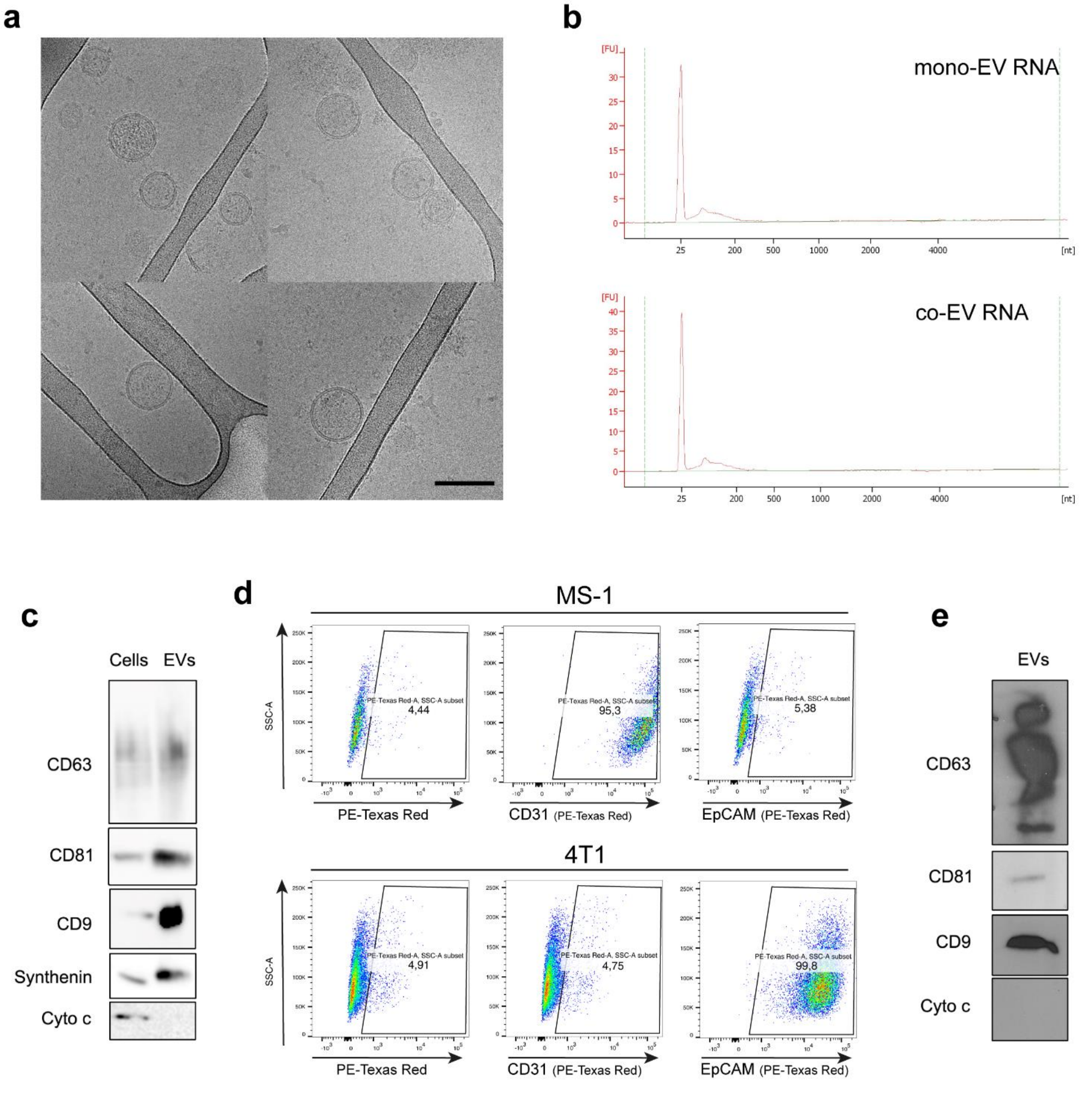

Supplementary Figure 2. EV and mouse cell characterization. a, Characterization of HUVEC EVs by cryo-transmission electron microscopy analysis bar represents 100 nm. Multiple images are shown. **b,** Bioanalyzer analysis of total RNAs extracted from mono- and co-EVs. There is an enrichment of small RNA fragments. **c**, Characterization of MS1 EVs by western blotting of the exosomal markers CD9, CD63, CD81 and syntenin in lysates from purified EVs and cells. Cyto c is a negative control. **d**, Flow cytometry analysis of MS-1 and 4T1 cells stained for the endothelial marker CD31 or EpCAM. Left panels are controls with PE-secondary antibody. **e**, Characterization of EVs isolated from mice with CD31 beads by western blotting of the exosomal markers CD9, CD63 and CD81 in lysates from purified EVs and cells. Cyto c is a negative control.

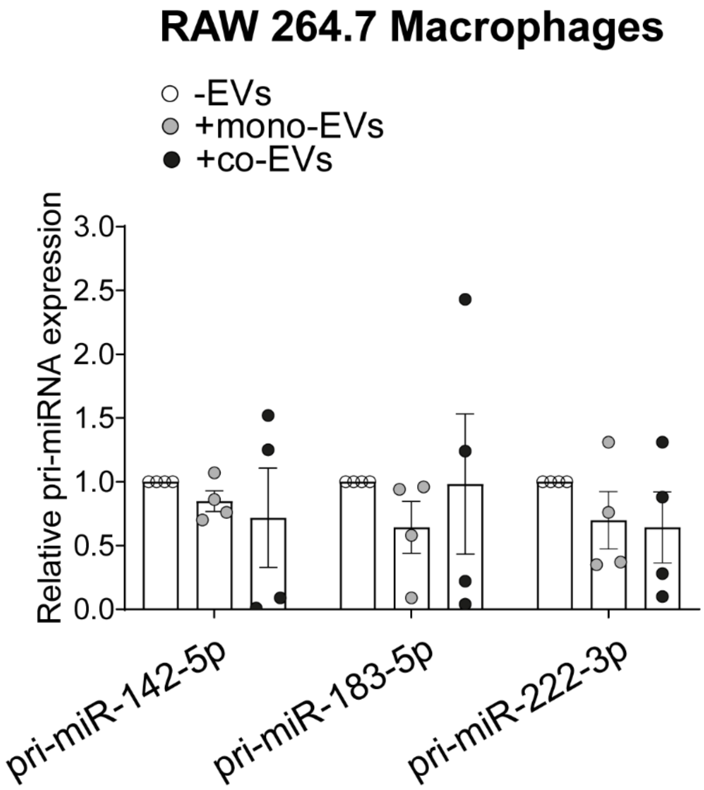

Supplementary Figure 3. Relative levels of precursors pri/pre-miR-142-5p, pri-pre-miR-183-5p and pri-pre-miR-222-3p after treatment of RAW 264.7 cells with mono- and co-EVs compared to no EV treatment, assessed by RT-qPCR. Data are presented as mean ± SEM. relative to the respective controls.

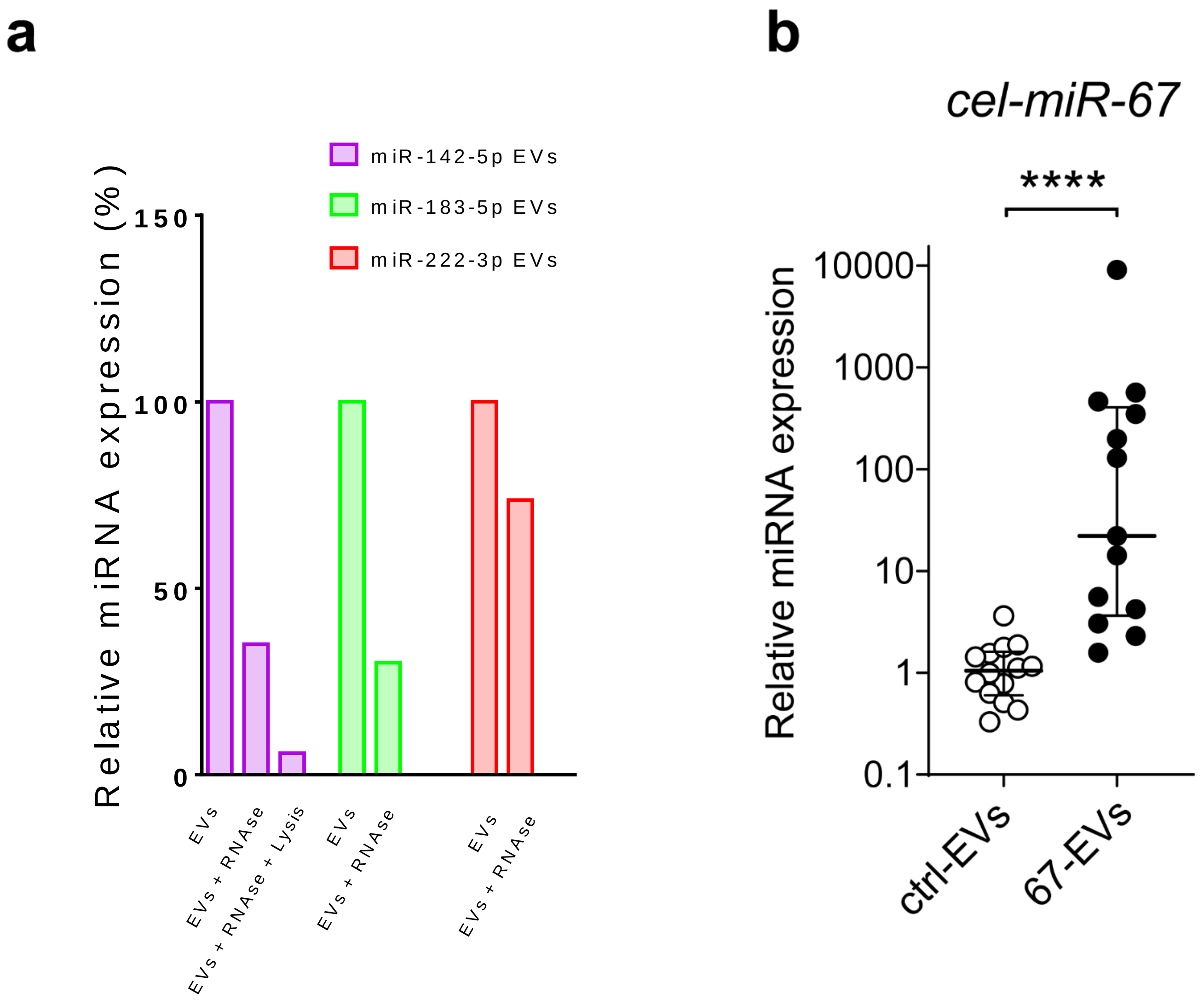

Supplementary Figure 4. Validation of the use of eletroporated EVs a,. Treatment of purified EVs by RNAse. EVs purified from MS-1 and electroporated with miR-142-5p, miR-183-5p and miR-222-5p. 10 µg of EVs were treated with or without RNase A and/or lysis buffer containing Triton X-100 and SDS before RNA extraction. **b**, **Relative levels of cel-miR-67 in tumors from mice injected with EVs loaded or not with cel-miR-67 mimic.** Validation of the use of EVs to transfer miRNA mimic into tumors by injecting MS-1 endothelial EVs loaded with exogenous miRNA (cel-miR-67 mimic) in the peritumoral region of mice harboring 4T1 tumors. Data are presented as median ± IQR. ****p <0.0001 calculated by unpaired two-sided Mann-Whitney test. Mice treated with ctrl-EVs, n=14 biologically independent samples; mice treated with miR-67-EVs, n=13 biologically independent samples.

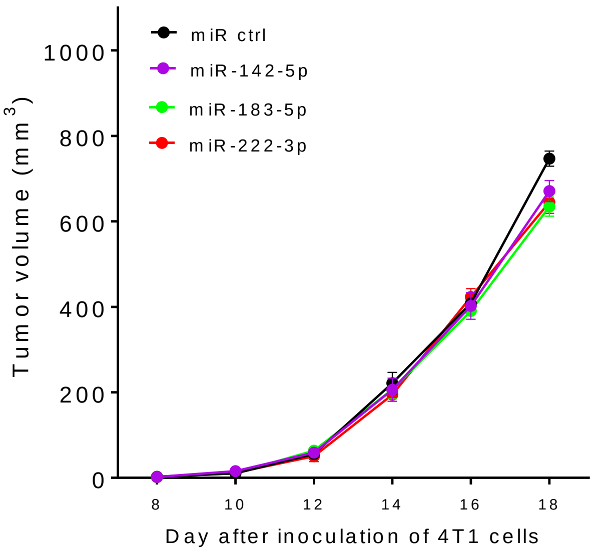

Supplementary Figure 5: miR-142-5p, miR-183-5p and miR-222-3p miR mimics do not increase tumor growth in a murine model of breast cancer. Kinetics of tumor growth in mice treated with miR-142-5p, miR-183-5p or miR-222-3p compared with those treated with cel-miR-67(ctrl). All data are not significantly different calculated by two-way ANOVA with Bonefrroni's test. Mice treated with cel-miR-67(ctrl)-EVs (n=4 mice), miR-142-5p-EVs (n=5 mice), miR-183-5p-EVs (n=5 mice) or miR-222-3p-EVs (n=5 mice), and data shown are from one experiment.

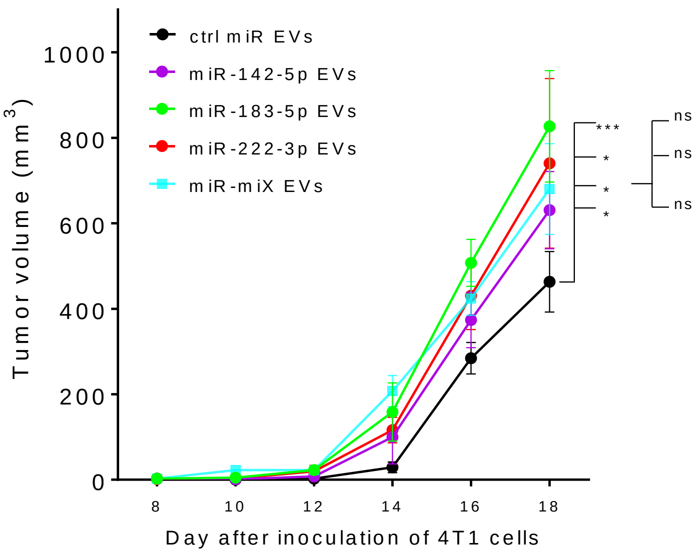

Supplementary Figure 6: Combination of miR-EVs (miR-miX) does not increase tumor growth compared to miR-142-5p-EVs, miR-183-5p-EVs and miR-222-3p-EVs alone. Kinetics of tumor growth in mice injected with 4T1 cells and treated with miR-142-5p-EVs, miR-183-5p-EVs, miR-222-3p-EVs or a combination of the three (or miR-miX:light blue) compared with those treated with cel-miR-67(ctrl). *p <0.05 ***p <0.001 calculated by two-way ANOVA with Bonferroni's test. 4 mice per group. Data shown are from one experiment.

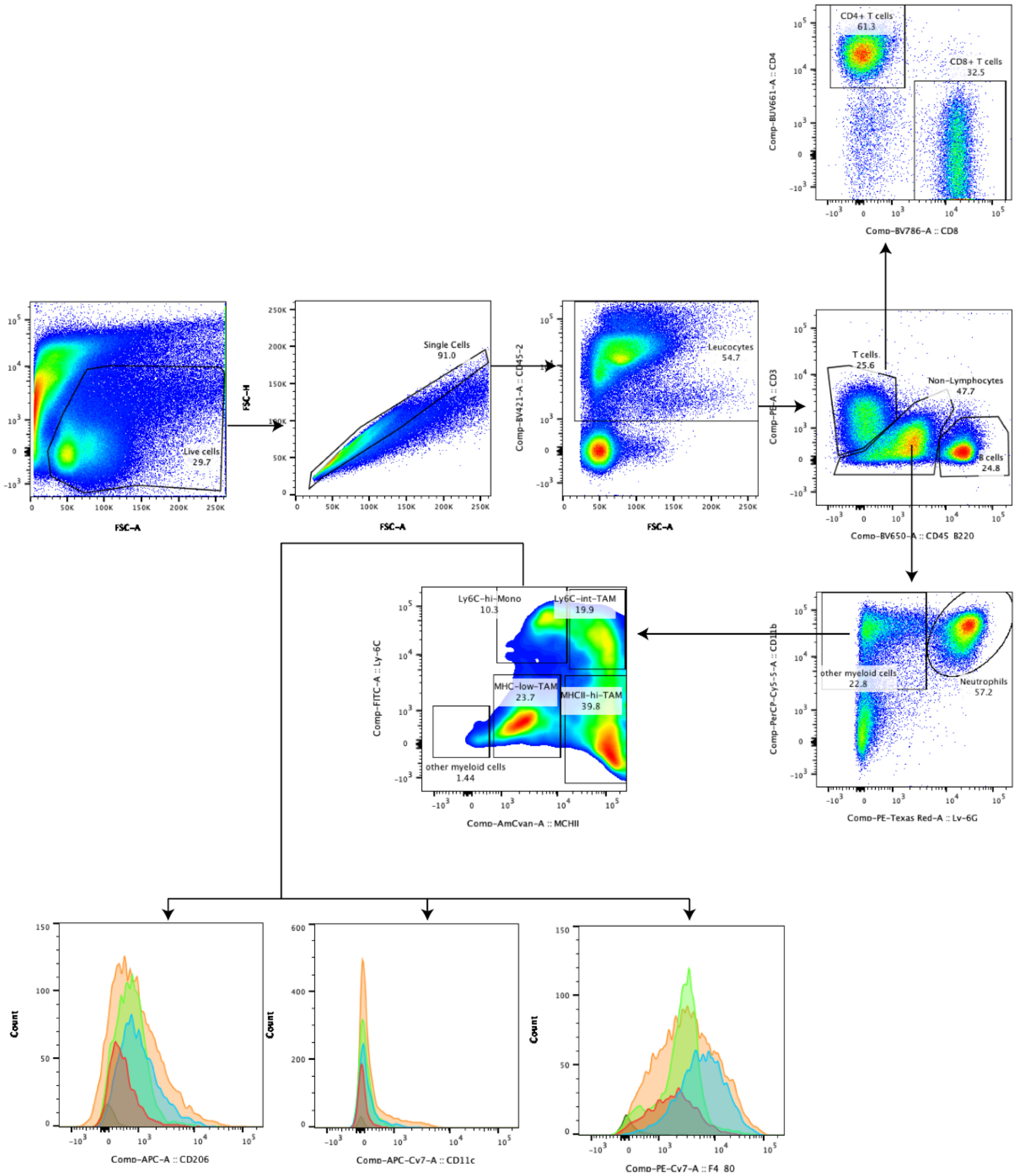

Subset Name	Mean : Comp-APC-A
Ly6C-hi-Mono	722
Ly6C-int-TAM	1631
MHC-low-TAM	989
MHCII-hi-TAM	1891
other myeloid cells	127

Subset Name	Mean : Comp-APC-Cy7-A
Ly6C-hi-Mono	92.9
Ly6C-int-TAM	248
MHC-low-TAM	164
MHCII-hi-TAM	467
other myeloid cells	37.7

Subset Name	Mean : Comp-PE-Cy7-A
Ly6C-int-TAM	8890
Ly6C-hi-Mono	2682
MHC-low-TAM	3252
MHCII-hi-TAM	6735
other myeloid cells	311

Supplementary Figure 7. Gating strategy for immune profile analyses. Briefly, single-cell suspensions of mouse 4T1-tumor were stained with using PE-anti-CD3e, BV421-anti-CD45.2, BUV395-anti-NK.1.1, APC-Cy7-anti-CD11c, AmCyan-anti-MHC-II, BUV661-anti-CD4, BV786-anti-CD8, FITC-anti-Ly-6C, APC-anti-CD206, PE-Cy7-anti-F4/80, PE-TexasRed-anti-Ly-6G, PerCP-Cy5.5-anti-CD11b all together with viability dye FVs510. Singlets were selected bases on FSC-H and FSC-A (out of live cells). Immune cells were next selected based on the expression of CD45 cell surface marker. B (B220+)and total T cells (CD3+) were gated out of CD45+ cells. T cells were further subdivided into CD8+ and CD4+ T cells using anti-CD4 and anti-CD8 antibodies. CD11b+Ly6G+ cells (gated out of non-lymphocytes (CD45+/CD3-/B220-)) were considered as neutrophils. The rest of myeloid cells (CD11b+/Ly6G-) were further subdivided into the following subsets based on MHCII and Ly6C intensity of staining: 1) Ly6C high (hi)/MHCII-low/neg; 2) Ly6C-intermediate (int)/MHCII-high (Ly6C-int-TAM); 3) MHC-low-Ly6C-low (MHC-low-TAM); 4) MHCII-high (hi)/Ly6C-low/neg (MHCII-hi-TAM) and 5) MHCII-neg/Ly6Cneg). Each of these 5 subpopulations was next analysed for surface expression using anti-F4/80, anti-CD11c and anti-CD206. For these 3 later markers, data are presented as a mean fluorescence intensity (MFI) for each of the 5 myeloid cells populations.

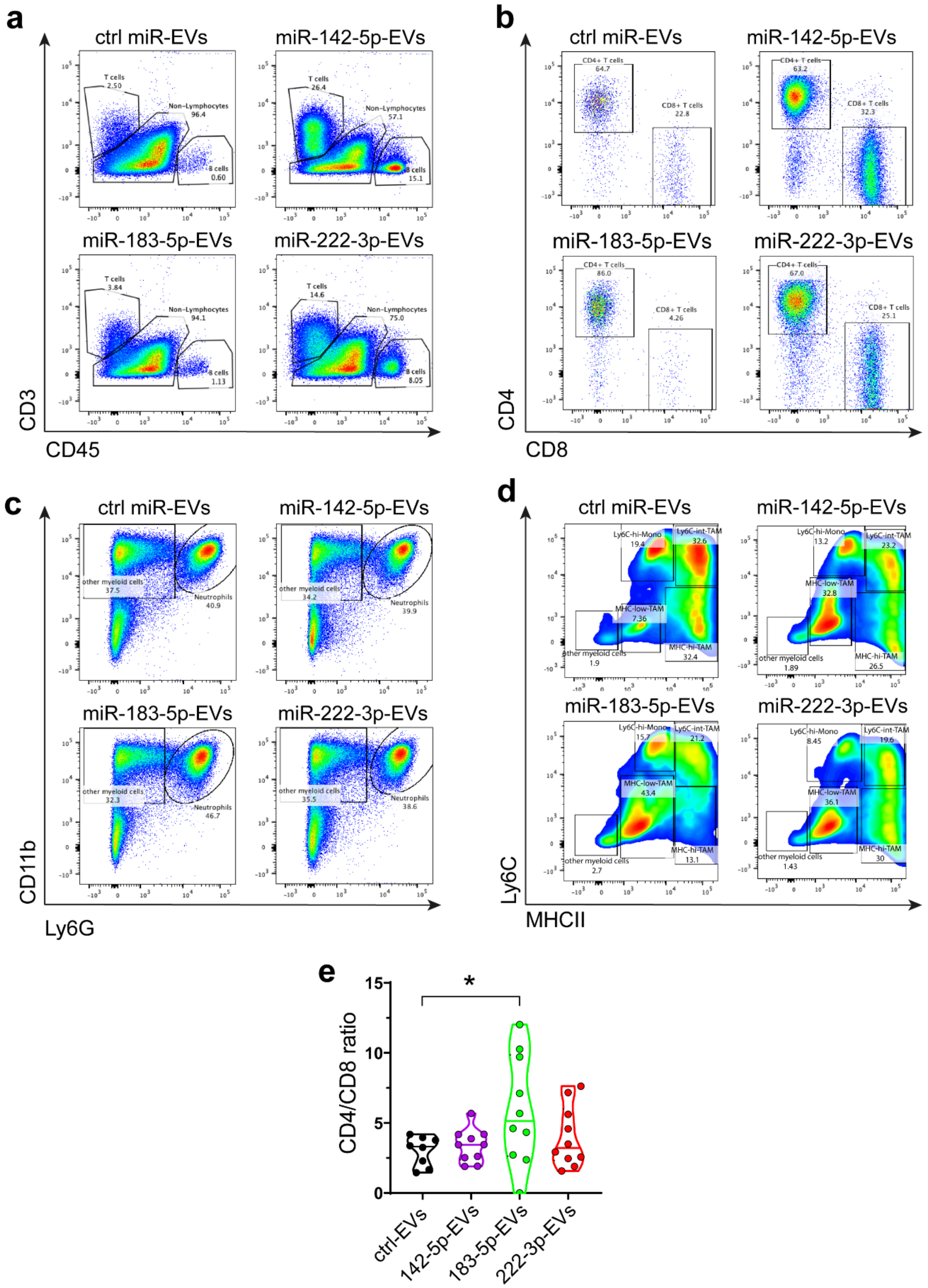

Supplementary Figure 8. Flow cytometry data from 4T1-tumor bearing mice treated with cel-miR-67 (ctrl)-EVs, miR-142-5p-EVs, miR-183-5p-EVs and miR-222-3p-EVs. Flow cytometry analysis as described in figure S5 (**a**), CD8+ and CD4+ expression in T lymphocytes (by gating CD3+ population) (**b**), neutrophils (Ly6G+ on CD45+ cells) (**c**) and TAM subpopulations (**d**) from 4T1-tumor bearing mice treated with cel-miR-67 (ctrl)-EVs, miR-142-5p-EVs, miR-183-5p-EVs and miR-222-3p-EVs. **e**, Ratio of CD4/CD8 of T cells in 4T1-tumor bearing mice treated with cel-miR-67 (ctrl)-EVs, miR-142-5p-EVs, miR-183-5p-EVs and miR-222-3p-EVs. Bars represent median values (violin plot). *p <0.05 calculated by ordinary one-way ANOVA followed by Dunnett correction. Mice treated with cel-miR-67(ctrl)-EVs (n=8 mice), miR-142-5p-EVs (n=10 mice), miR-183-5p-EVs (n=10 mice) or miR-222-3p-EVs (n=10 mice), and data shown are from two independent experiments.

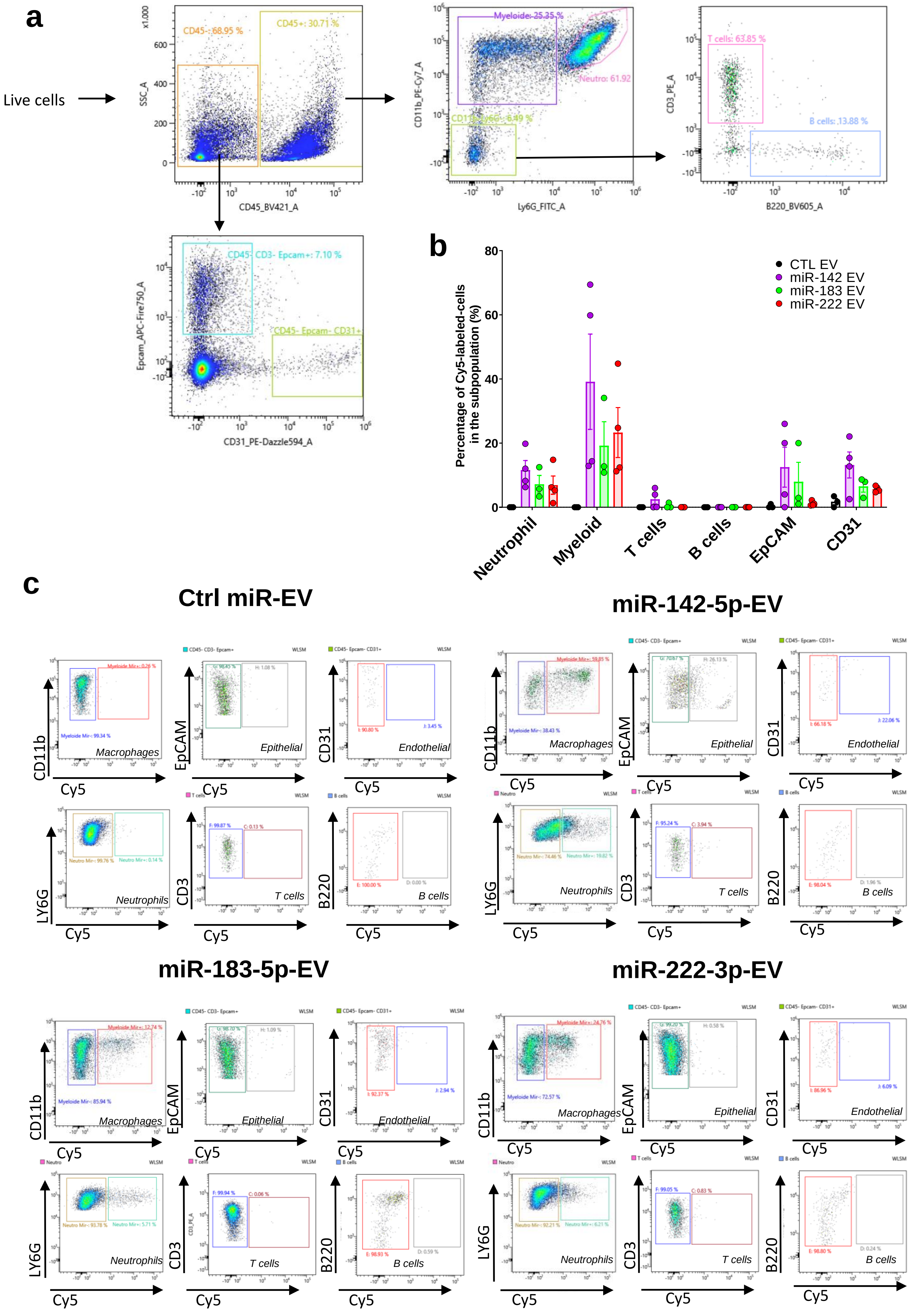

Supplementary Figure 9 –legend next page

Supplementary Figure 9. Flow cytometry data from 4T1-tumor bearing mice treated with Cy-5 labelled miRs in cel-miR-67 (ctrl)-EVs, miR-142-5p-EVs, miR-183-5p-EVs and miR-222-3p-EVs. a, **Gating strategy.** Briefly, single-cell suspensions of mouse 4T1-tumors were stained with using PE-anti-CD3e, BV421-anti-CD45.2, BUV395-anti-NK.1.1, APC-Cy7-anti-CD11c, AmCyan-anti-MHC-II, BUV661-anti-CD4, BV786-anti-CD8, FITC-anti-Ly-6C, APC-anti-CD206, PE-Cy7-anti-F4/80, PE-TexasRed-anti-Ly-6G, PerCP-Cy5.5-anti-CD11b all together with viability dye FVs510. Singlets were selected based on FSC-H and FSC-A (out of live cells). Non immune cells were gated out the CD45- cells CD31+ cells were considered as endothelial cells. EpCam+ cells were considered as epithelial cells and represent the cancer cell population. Immune cells were next selected based on the expression of CD45 cell surface marker. B (B220+) and total T cells (CD3+) were gated out of CD45+/CD11b-/Ly6G- cells. CD11b+/Ly6G+ cells were considered as neutrophils. CD11b+/Ly6G- cells were considered as myeloid cells (CD11b+/Ly6G-). Each population was analyzed for the presence of the Cy5-labeled miR using PE. b, Flow cytometry analysis of 4T1 tumors from mice treated with every other day with cel-miR-67 (ctrl)-EVs, miR-142-5p-EVs, miR-183-5p-EVs and miR-222-3p-EVs and with Cy5-labeled miRs in EVs the day before sacrifice. Ctrl mice were treated with unlabeled cel67-miR EVs. Analysis of the presence of Cy5-miRs in the following subpopulations: neutrophils, endothelial, epithelial, B and T cells. Panels for macrophages are shown in main figure 4. Bars represent mean +SEM. *p <0.05 calculated by ordinary one-way ANOVA followed by Dunnett correction. Mice treated with cel-miR-67(ctrl)-EVs (n=4 mice), miR-142-5p-EVs (n=4 mice), miR-183-5p-EVs (n=3 mice) or miR-222-3p-EVs (n=4 mice), and data shown are from two independent experiments.

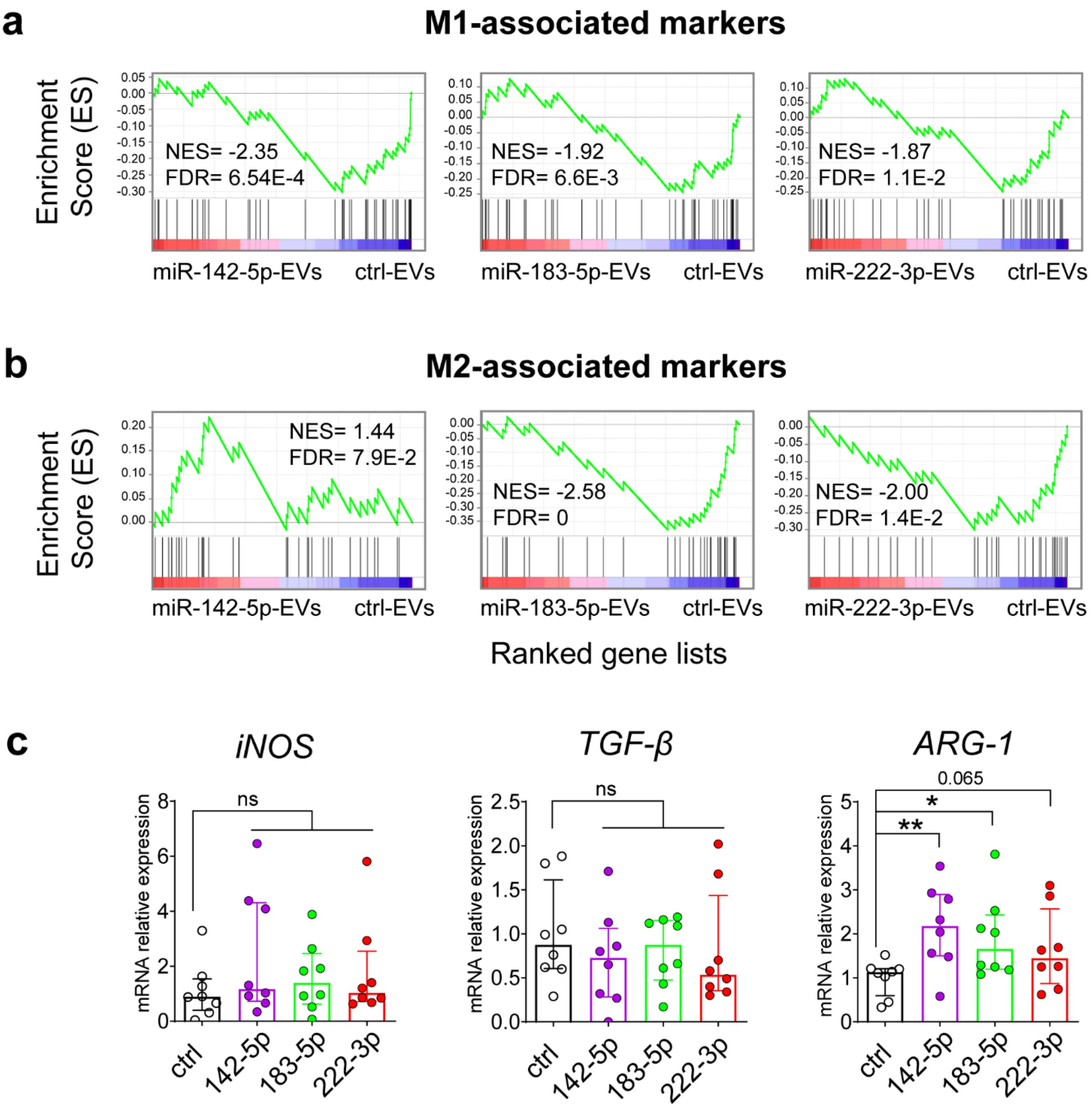

Supplementary Figure 10. GSEA analysis from 4T1-tumor bearing mice treated with cel-miR-67 (ctrl)-EVs, miR-142-5p-EVs, miR-183-5p-EVs and miR-222-3p-EVs. GSEA analysis of enrichment of (**a**) M1- and (**b**) M2- associated markers in 4T1-tumors treated with miR-142-5p-EVs, miR-183-5p-EVs or miR-222-3p-EVs compared to those treated with cel-miR-67 (ctrl)-EVs. (**c**) RT-qPCR was used to assess the impact of miR-142-5p, miR-183-5p and miR-222-3p on mRNA expression of iNOS, TGF-β and ARG-1 in cells from mouse 4T1-tumors. Data are presented as median ± IQR. *p <0.05 and **p <0.01 calculated by Kruskal-Wallis test with Dunn correction. Mice treated with cel-miR-67(ctrl)-EVs (n=8 mice), miR-142-5p-EVs (n=8 mice), with miR-183-5p-EVs (n=8 mice) and with miR-222-3p-EVs (n=8 mice).

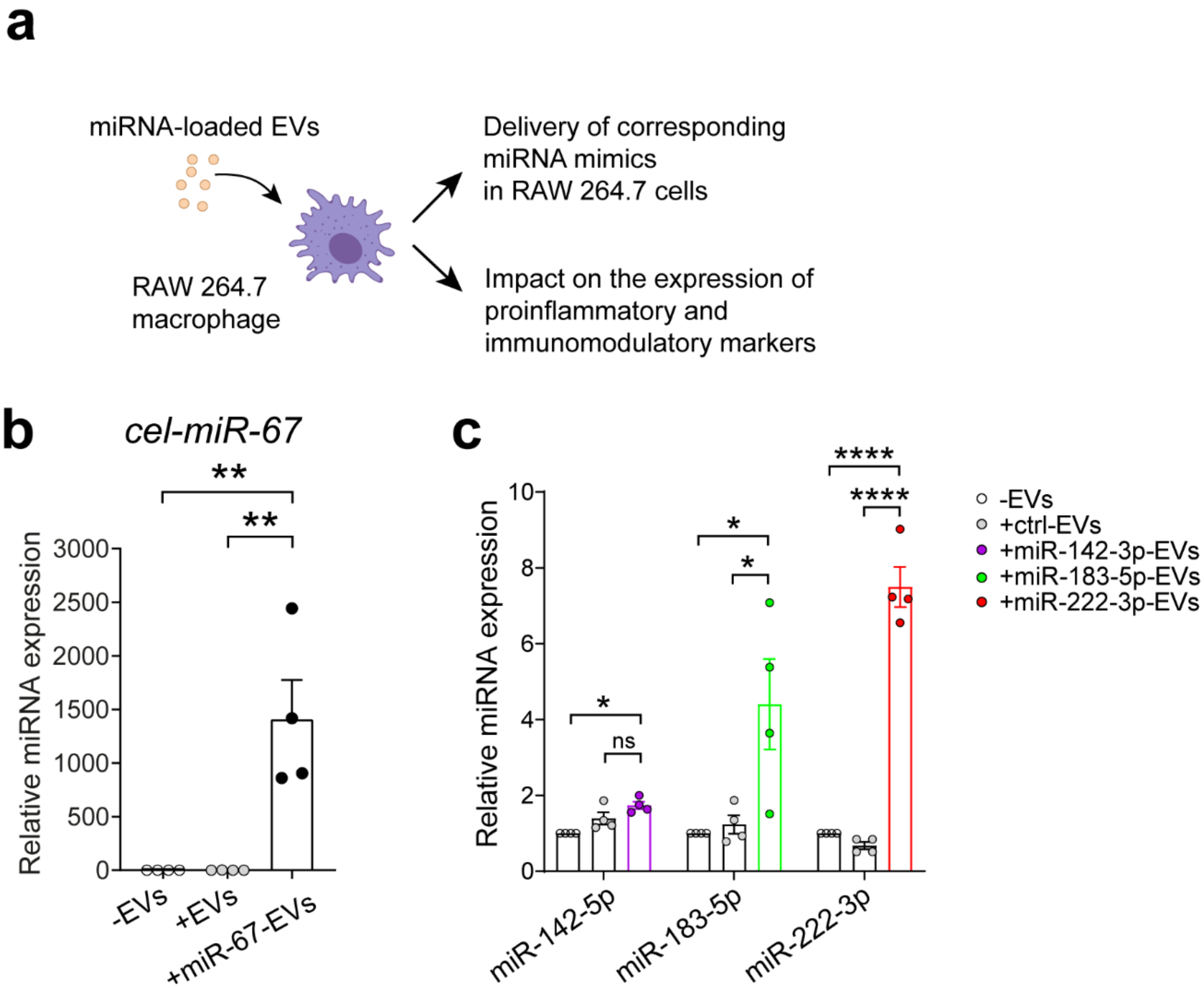

Supplementary Figure 11. Transfer of miRNA mimics in RAW 264.7 macrophages via miRNA-loaded EVs. **a,** Overview of the treatment of RAW 264.7 macrophages with miRNA-loaded EVs. MiR-loaded EVs were added to RAW 264.7 macrophages for 24 h, then the delivery of miRNA mimics as well as their impact on the expression of M1 and M2 markers were assessed. **b,c**, Relative levels of cel-miR-67 (**b**) and candidate miRNAs (miR-142-5p, miR-183-5p and miR-222-3p) (**c**) in RAW 264.7 cells treated with corresponding miRNA-loaded EVs, assessed by RT-qPCR (n=4). RNA levels are expressed relative to the respective controls (-EVs bars).*p <0.05, **p <0.01, ****p <0.0001 calculated by RM one-way ANOVA with Tukey's test (n=4).

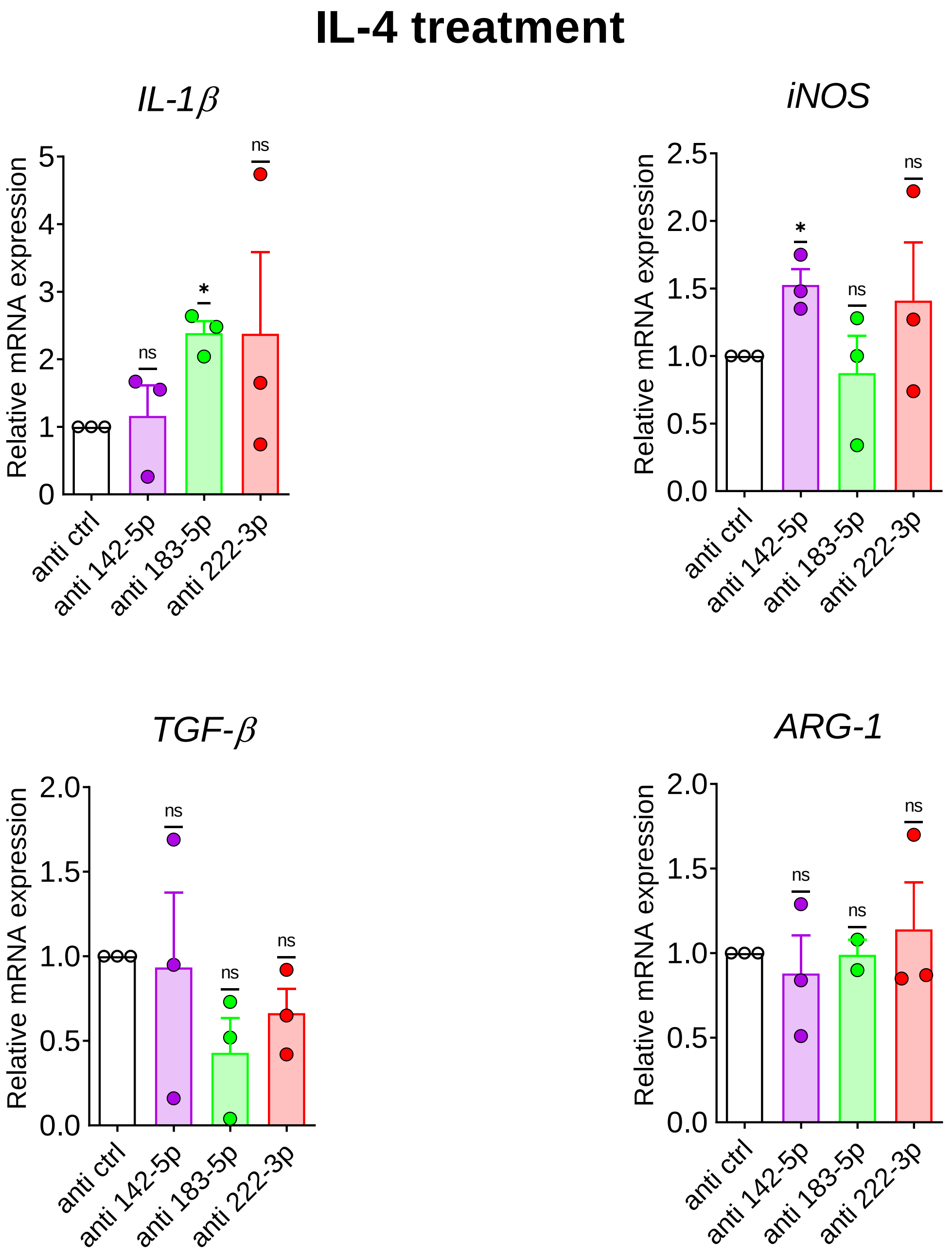

Supplementary Figure 12. Anti-miR transfection in RAW 264.7 macrophages. a, , miR-142-5p, miR-183-5p and miR-222-3p anti-miRs were transfected in RAW 264.7 macrophages. 24h later cells were washed and treated with interleukine 4 for 48h. The expression of M1 (IL-1β, iNOS) and M2 markers (TGF-β, ARG-1) was assessed by RT-qPCR. RNA levels are expressed relative to the respective controls (-EVs bars).*$p < 0.05$, calculated by RM one-way ANOVA with Tukey's test (n=3).

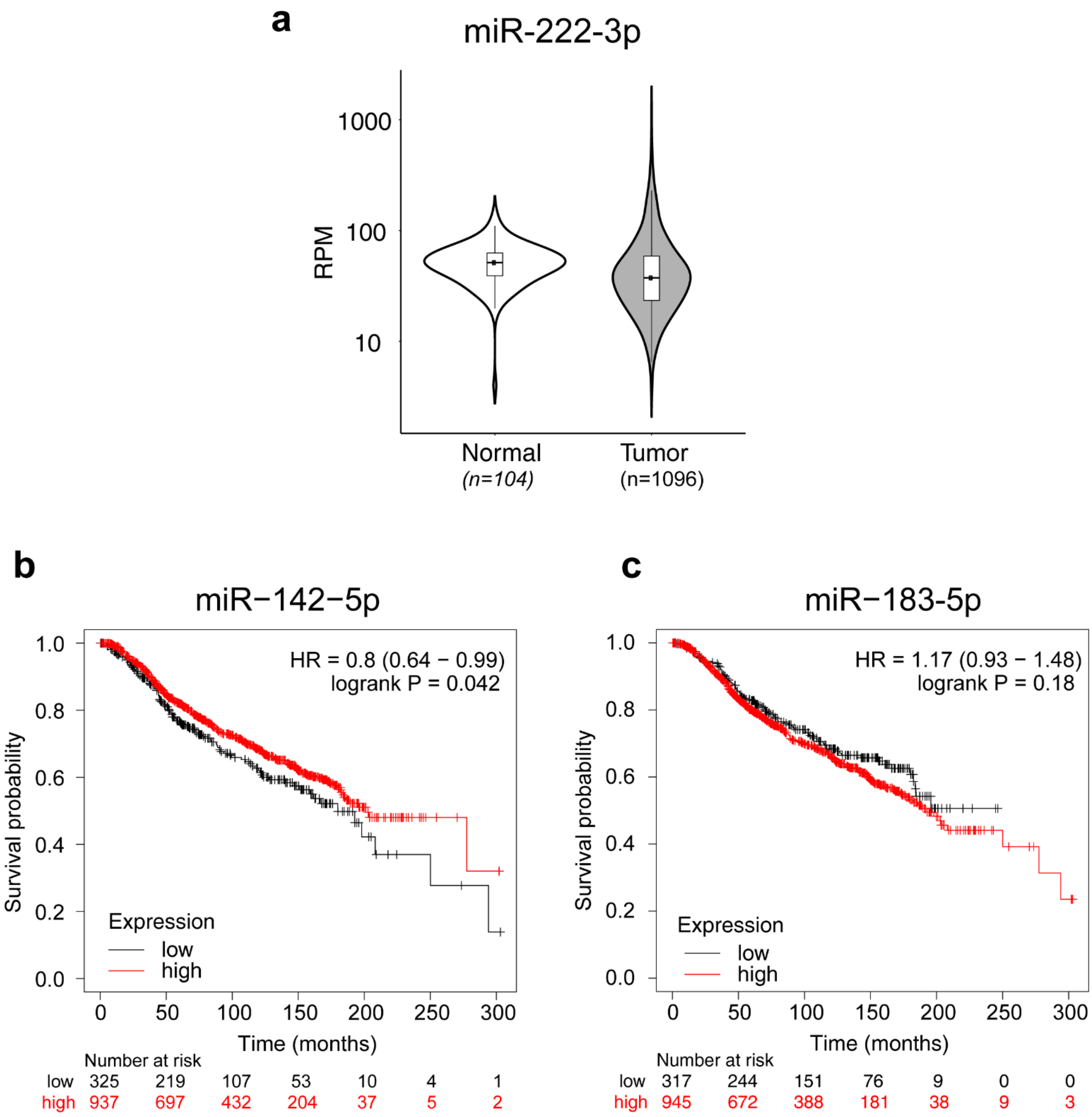

Supplementary Figure 13: MiR-222-3p level in breast cancer patients and survival curves for miR-142-5p ad miR-183-5p

a, Levels of miR-222-3p in normal tissue and tumors from breast cancer patients. RPM = RNA-Seq reads per million mapped reads. **b**, Kaplan-Meier survival plot of breast cancer patients with high vs. low levels of miR-142-5p. Expression range of the probe: 6 - 13, cutoff value: 7.88. **c**, Kaplan-Meier survival plot of breast cancer patients with high vs. low levels of miR-183. Expression range of the probe: 6 - 14, cutoff value: 9.31.
