# Supplementary material for: Endothelial extracellular vesicles promote tumour growth by tumour‐associated macrophage reprogramming
Source: J Extracell Vesicles. 2022 Jun 3;11(6):e12228. doi: 10.1002/jev2.12228 (PMC9164145; doi:10.1002/jev2.12228)
Supplement: Supplementary file 4 — Supporting Information [file JEV2-11-e12228-s001.pdf]

Supplementary Tables

Supplementary Table 3: List of primers used for qRT-PCR.

Gene	Forward Primer (5'->3')	Reverse Primer (5'->3')
hsa miR-429	GCTAATACTGTCTGGTAAAACCGTAA	Universal reverse primer
hsa miR-142-5p	CGCCATAAAGTAGAAAGCACTACTAA	Universal reverse primer
hsa miR-183-5p	CGCCTATGGCACTGGTAGAA	Universal reverse primer
hsa miR-138-5p	AGCTGGTGTTGTGAATCAGGCCG	Universal reverse primer
hsa miR-222-3p	GCTACATCTGGCTACTGGGTAAA	Universal reverse primer
hsa miR-4488	GGGCTCCGGCGAAAA	Universal reverse primer
hsa miR-4532	GGAGCCCGGCGAAAA	Universal reverse primer
hsa miR-146a-5p	TGAGAACTGAATTCCATGGGTTA	Universal reverse primer
hsa miR-1246	AATGGATTTTTGGAGCAGG	Universal reverse primer
hsa miR-100-5p	AACCCGTAGATCCGAACTTGTG	Universal reverse primer
hsa miR-29a-3p	GCACCATCTGAAATCGGTTAAA	Universal reverse primer
hsa miR-210-3p	TGTGACAGCGGCTGAAAA	Universal reverse primer
hsa miR-378a-3p	ACTGGACTTGGAGTCAGAAGG	Universal reverse primer
hsa miR-1290	TGGATTTTTGGATCAGGGA	Universal reverse primer
hsa miR-4792	AGCGCTCGCTGGCAAAA	Universal reverse primer
hsa miR-7-5p	CGTGGAAGACTAGTGATTTTGTTG	Universal reverse primer
hsa miR-326	CCTCTGGGCCCTTCCTCCAG	Universal reverse primer
hsa miR-335-5p	TCAAGAGCAATAACGAAAAATGT	Universal reverse primer
hsa miR-127-3p	GATCCGTCTGAGCTTGGCTA	Universal reverse primer
hsa miR-376c-3p	GAACATAGAGGAAATTCCACGTAAA	Universal reverse primer
hsa miR-3651	CGGTCGCTGGTACATGAAA	Universal reverse primer
hsa-let-7a-5p	TGAGGTAGTAGGTTGTATAGTT	Universal reverse primer
hsa miR-26a-5p	TTCAAGTAATCCAGGATAGGCTAAA	Universal reverse primer
hsa miR-191-5p	CAACGGAATCCCAAAAGCAGCTG	Universal reverse primer
hsa miR-16	TAGCAGCACGTAAATATTGGCG	Universal reverse primer
hsa U6 snRNA	CGCAAGGATGACACGCAAATTC	Universal reverse primer
hsa miR-191-5p	CAACGGAATCCCAAAAGCAGCTG	Universal reverse primer
cel miR-67-5p	TCACAACCTCCTAGAAAGAGTAGA	Universal reverse primer
mmu-U6	TGGCCCCTGCGCAAGGATG	Universal reverse primer

mmu pri/pre-miR-142-5p	TATTTCATCTTTCGTGATGATTGTC	CCTTTGTGATGTGGGAGGT
mmu pri/pre-miR-183-5p	CATACCGTGACCATCTTAAGTGAC	CAAATACCGGGAAGCCATT
mmu pri/pre-miR-222-3p	ACCGAGTCATCGGTCACAT	CTCTGGGTCATCGGTCTACA
mmu IL-1β	AGTTGACGGACCCCAAAAGA	TGCTGCTGCGAGATTTGAAG
mmu iNOS	TCATTGGGCCTGGTACGGGCA	ACACCAAGCTCATGCGGCCTC
mmu TGF-β1	GAGAGCCCTGGATACCAACT	CAACCCAGGTCCTTCCTAAA
mmu ARG-1	TGGGCAACCTGTGTCCTTTCTCCT	TTCCCCAGGGTCTACGTCTCGCA

Supplementary Table 4: List of miRNA mimics and antimiRs. Cy5-labeled miRs are the same duplexes but with a Cy5 at the 5' end of the sens strand.

Gene	Sens	AntiSens
mmu-miR-142-5p	5' P-CAUAAAGUAGAAAGCACUACU 3'	5' UAGUGCUUUAUACUCUAUAUU 3'
mmu-miR-183-5p	5' P-UAUGGCACUGGUAGAAUUCACU 3'	5' UGAAUUCUACAAGUGUCAUUUU 3'
mmu-miR-222-3p	5' P- AGCUACAUCUGGCUACUGGGU 3'	5' CCAGUAGCCGGAUAUAGCCUU 3'
cel-miR-67-3P	5' P-UCACAACCUCCUAGAAAGAGUAGA 3'	5' UACUCUUUCUAGAAGGUGGUGCUU 3'

Supplementary Table 5: List of anti-miRs. Anti-miRs are made with complete phosphorotioate backbone with a mix of DNA and LNA bases. LNAs are in the brackets.

Gene	Anti-miRs
mmu-miR-142-5p	5'(AG)TAGT(G)CTT(T)CT(A)CTTT(ATG) 3'
mmu-miR-183-5p	5'(AG)TGAA(T)TCT(A)CCA(G)TGCC(ATA) 3'
mmu-miR-222-3p	5'(AC)CCAG(T)AGC(C)AGAT(G)TA(GCT) 3'
cel-miR-67-3P	5'(TC)GATCTA(C)TCTTTCTAGG(A)GGTTG(T)GATG(CT) 3'
